# Spotlight on Differentially Expressed Genes in Urinary Bladder Cancer

**DOI:** 10.1371/journal.pone.0018255

**Published:** 2011-04-05

**Authors:** Apostolos Zaravinos, George I. Lambrou, Dimitrios Volanis, Dimitris Delakas, Demetrios A. Spandidos

**Affiliations:** 1 Laboratory of Virology, Medical School, University of Crete, Heraklion, Crete, Greece; 2 First Department of Pediatrics, Choremeio Research Laboratory, University of Athens, Athens, Greece; 3 Department of Urology, Asklipieio General Hospital, Voula, Athens, Greece; Georgia Institute of Technology, United States of America

## Abstract

**Introduction:**

We previously identified common differentially expressed (DE) genes in bladder cancer (BC). In the present study we analyzed in depth, the expression of several groups of these DE genes.

**Materials and Methods:**

Samples from 30 human BCs and their adjacent normal tissues were analyzed by whole genome cDNA microarrays, qRT-PCR and Western blotting. Our attention was focused on cell-cycle control and DNA damage repair genes, genes related to apoptosis, signal transduction, angiogenesis, as well as cellular proliferation, invasion and metastasis. Four publicly available GEO Datasets were further analyzed, and the expression data of the genes of interest (GOIs) were compared to those of the present study. The relationship among the GOI was also investigated. GO and KEGG molecular pathway analysis was performed to identify possible enrichment of genes with specific biological themes.

**Results:**

Unsupervised cluster analysis of DNA microarray data revealed a clear distinction in BC vs. control samples and low vs. high grade tumors. Genes with at least 2-fold differential expression in BC vs. controls, as well as in non-muscle invasive vs. muscle invasive tumors and in low vs. high grade tumors, were identified and ranked. Specific attention was paid to the changes in osteopontin (OPN, SPP1) expression, due to its multiple biological functions. Similarly, genes exhibiting equal or low expression in BC vs. the controls were scored. Significant pair-wise correlations in gene expression were scored. GO analysis revealed the multi-facet character of the GOIs, since they participate in a variety of mechanisms, including cell proliferation, cell death, metabolism, cell shape, and cytoskeletal re-organization. KEGG analysis revealed that the most significant pathway was that of Bladder Cancer (p = 1.5×10^−31^).

**Conclusions:**

The present work adds to the current knowledge on molecular signature identification of BC. Such works should progress in order to gain more insight into disease molecular mechanisms.

## Introduction

Urinary bladder cancer (BC) is the most common malignancy of the urinary tract, responsible for significant mortality and morbidity worldwide. In Europe, an estimated 105,000 new cases of bladder cancer are diagnosed annually, while approximately 30,000 patients die from bladder cancer each year [1]. Its incidence directly increases with age, being rare before the age of 50, and it is three to four times more common in men compared to women. Nearly all bladder cancers are carcinomas, arising from the transitional epithelium. The behaviour of transitional cell carcinoma (TCC) is highly diverse and defined by two separate, but related processes: tumor recurrence and progression. At presentation, 75–85% of tumors are restricted to the mucosa, or invade the *lamina propria mucosae*. The remainder present with invasion of the muscular layer of the bladder wall or extend to perivesical tissue, adjacent organs and the pelvic wall. More than 60% of the superficial tumors will recur at least once and progress to less differentiated or invasive neoplasms with worse prognosis in a significant percentage of patients [2]. The most useful prognostic parameters are tumor grade, stage, size, prior recurrence rate and the synchronous presence of CIS [3]. Nevertheless, better understanding of the natural history of TCC can be expected upon the elucidation of the molecular mechanisms of TCC.

We previously identified common differentially expressed (DE) genes in urinary BC [4]. In the present study, our interest was focused on the analysis of various groups of DE genes, such as genes involved in the control of the cell cycle, DNA damage repair, apoptosis, signal transduction, transcription factors, angiogenesis, cellular proliferation, invasion and metastasis. We explored the expression profile in BC vs. healthy tissue, in non-muscle-invasive (stage T1) vs. muscle-invasive tumors (stage T2-T4), and in low vs. high grade tumors by microarray analysis. Our results were further validated by immunohistochemistry, qPCR and Western blotting. Moreover, we performed GEO computational analysis in microarray data extracted from other publicly available datasets, and compared them with our results.

Our results focused on genes which play a significant role in the most important cellular processes and demonstrated a large difference in their expression patterns between BC and control samples. Genes with at least 2-fold differential expression in BC vs. controls, as well as in non-muscle-invasive vs. muscle-invasive tumors, and in low vs. high grade tumors, were identified and ranked.

## Materials and Methods

### Study design and clinicopathological data

Paired tumor and normal tissue samples from a consecutive series of 30 patients with newly diagnosed BCs undergoing transurethral bladder tumor resection at the Department of Urology, “Asklipieio” General Hospital, Athens, were acquired after the amount of tissue necessary for routine pathology examination had been removed (**Table 1**). All tumor specimens were classified and graded by the same pathologist. Cancer patients were categorized accordingly into muscle-invasive (T2, T3 or T4) and non-invasive tumors (Ta, T1, and CIS). For comparative purposes with previous reports, the 1973 World Health Organization (WHO) grading system [low grade (grades 1–2) and high grade (grade 3)] was used in this study which is still the most commonly used system despite being superseded by the 2004 WHO/International Society of Urologic Pathology (ISUP) classifications [5]. Written informed consent was obtained from all patients included in this study. The study protocol was approved by the Ethics Committee of the University of Crete. Eligibility criteria used were electively resected primary BCs and the availability of DNA from normal and tumor tissue for biomolecular analyses. Exclusion criteria were a history of previous neoplasms and chemotherapy or radiation therapy prior to surgery.

**Table 1:** Clinicopathological characteristics of the patients.

Subjects (n)	30*
Sex	
Male	27
Female	3
Age (years)	
mean	72.2
range	44–86
Smoke†	
NS	4
FS	8
S	18
Occupational exposure‡	
yes	19
no	11
Stage	
Ta	1
T1	12
T1a	4
T1b	1
T2	2
T2a	1
T2b	5
T2+in situ	3
T3a	1
Grade (WHO 1973)	
I	0
II	10
III	20
Grade (WHO/ISUP 2004)	
Low	8
High	22

*30 TCCs and 30 adjacent normal tissue.

† NS, non-smoker; FS, former smoker; S, smoker.

‡ Exposure to chemicals, paints, pesticides, petroleum, ink, etc.

Tissue samples were obtained at surgery from the tumor and the following three grossly normal selected sites (cold cup biopsies): posterior wall, trigone, and the area adjacent to the tumor. Parts of the resected normal samples were sent for histopathological analysis. Tumor and normal tissues were frozen immediately in liquid nitrogen, transported and stored at −80°C until DNA extraction.

Patients with non-muscle-invasive BCs were followed up with periodical cystoscopic examinations and intravesical treatment as indicated. Patients with invasive BCs were offered radical cystectomy with or without systemic chemotherapy. After a mean follow-up of 24±3 months, 8 (26.6%) patients had recurrent tumors. In Ta/T1 tumors the frequency of recurrence was 29.4% (5/17) compared with 23% (3/13) of T2-T3 tumors. In patients with non-muscle-invasive BCs, the progression rate was 11.1% and 22.2% for grade 2 and grade 3 tumors, respectively. All recurrences were proven by biopsy.

### Immunohistochemistry

Sections, 3-mm thick, of formalin-fixed, paraffin-embedded tissue were cut and placed on slides coated with 3-aminopropyltriethoxysilone. Slides were dried at 56°C for 1 h before immunohistochemical staining. Tissue sections were deparaffinized in xylene before rehydration in graded alcohols, and endogenous peroxidase activity was blocked by treatment with 3% H_2_O_2_ at room temperature for 15 min. Antigen unmasking was performed by 30 min of incubation at 80°C in 10 mM trisodium citrate (pH 6.1). Immunostaining and revelation were performed on a Dako automate. Slides were incubated at room temperature with primary polyclonal goat antibodies against anti-ErbB2 (1∶800; Dako), cyclin D1 (1∶100; SP4; Epitomics), monoclonal antibody against anti-p53 (1∶250; E26; Epitomics) and monoclonal antibody against anti-Ki-67 (1∶100; SP6; Epitomics). Epitopes of the primary antibody were localized by the immunoperoxidase technique using the secondary antibody avidin-biotin complex and peroxidase substrate kit (kit 5001, Dako), according to the manufacturer's protocol. The sections were then treated with Chromagen 30-30 diaminobenzidene tetrahydrochloride to identify sites of immunoprecipitation by light microscopy. Finally, sections were washed, counterstained with hematoxylin, and mounted under coverslips. No specific staining was observed when the primary antibody was omitted from the protocol (negative control). Specificity of the immunostaining was additionally controlled by simultaneous staining of breast cancer samples with known ErbB2, cyclin D1, p53 and Ki67 expression patterns. An experienced pathologist scored the staining intensity at four levels (negative, weak, moderate and strong), considering both color intensity and number of stained cells. In brief, the proportion score represented the estimated percentage of positive-stained tumor cells (0 = 0%; 1 = 1%; 2 = 1 to 10%; 3 = 10 to 33%; 4 = 33 to 66%; 5≥ 67%), and the intensity score represented the depths of the tumor cell staining (1 = weakly positive; 2 = moderately positive; 3 = strongly positive).

### RNA purification and cDNA preparation

We isolated total RNA from crude tumor biopsy samples using a power homogenizer and the TRIzol® reagent (Invitrogen, Carlsbad, CA), as previously described [6], [7]. We used 2 µg total RNA as starting material for the cDNA preparation. We carried out the first and second-strand cDNA synthesis using the StrataScript First-Strand Synthesis System, as previously described [8].

### Microarray analysis

Oligos microarray chips (∼57K genes) were obtained from GE HealthCare (IL) and AppliedMicroarrays (MA) (CodeLink 57K Human Whole Genome). Hybridization was performed with the CodeLink RNA amplification and Labeling kit, utilizing the Cy5 fluorescent dye. Slides were scanned with a microarray scanner (ScanArray 4000XL). Images were generated with ScanArray microarray acquisition software (GSI Lumonics, USA). cRNAs from three experimental setups were used in single experiments with internal spikes as controls. The experimental setups consisted of 10 urinary BC samples of different histologies (T1/2-grade 3, T1-grade 1/2, T3-grade 3) and 5 control samples. The scanned images were further processed with the CodeLink Expression Analysis Software v5.0 from Amersham Biosciences. The experimental setup was analyzed based on the reference design as described previously [9], [10], [11]. All tumor samples were compared against the mean value of the control samples. Background correction was performed by subtracting the median global background from the median local background from the signal intensity. A threshold of 2 was set as cut-off, meaning that spot intensity for at least one channel should be twice as much as that of the background. Microarray data were normalized by dividing spot intensities by the global median. Normalized data were extracted, pre-processed and sorted with Microsoft Excel®. Array data are available at the Gene Expression Omnibus (National Center for Biotechnology Information) with accession numbers GSM678186 through GSM678385 (http://www.ncbi.nlm.nih.gov/geo/query/acc.cgi?acc=GSE27448). Furthermore, each gene was tested for its significance in differential expression using a z-test. Genes were considered to be significantly differentially expressed if they obtained a p-value <0.05. The False Discovery Rate was calculated as described previously [12], [13], [14]. Genes were further classified using two-way (genes-against samples) average-linkage hierarchical clustering with Euclidian distance using the Genesis 1.7.2 software (Technische Universitaet-Graz, Austria) [15].

### Real-time PCR validation

Transcribed products were subjected to real-time PCR assay with SYBR Green I in an Mx3000P programmable thermal controller apparatus (Stratagene, La Jolla, CA). The primer pairs were designed to span at least one intron in order to avoid amplification of the contaminating genomic DNA along with cDNA. Their sequence and the corresponding PCR product sizes are listed in **Table S1**. GAPDH and ACTB genes were used as internal controls [16].

One microliter of cDNA from normal or TCC samples, respectively, was amplified in a PCR reaction with 2x Brilliant SYBR® Green QPCR Master Mix (containing 2.5 mM MgCl_2_), 300 nM of each primer and 30 µM Rox passive reference dye in a final volume of 20 µl. After initial denaturation at 95°C for 10 min, samples were subjected to 40 amplification cycles comprising denaturation at 95°C for 30 sec, annealing at 60°C for 30 sec, and elongation at 72°C for 30 sec. Amplification and elongation steps were followed by a melt curve analysis in which the temperature was increased from 55°C to 95°C at a linear rate of 0.2°C/sec. Data collection was performed during both annealing and extension, with two measurements at each step and at all times during melt curve analysis. To verify the results of the melt curve analysis, qPCR products were analyzed by electrophoresis on 2% agarose gel, stained with ethidium bromide and photographed on a UV light transilluminator (**Figure S1**). In each qPCR reaction two negative controls were included, one with no cDNA template and one with no reverse transcription treatment. All samples were treated in duplicate. Gene transcription levels were calculated using the ΔΔCt method, as previously described [17], [18].

### Total protein extraction and Western blot analysis

After addition of 250 µl ice-cold GST-Fish lysis buffer (10% glycerol, 50 mM Tris (pH 7.4), 100 mM NaCl, 1% (v/v) Nonidet P-40, 2 mM MgCl_2_, and a protease inhibitor cocktail (Roche Diagnostics GmbH, Germany), the homogenized tissue samples were centrifuged at 14,000 x g for 15 min at 4°C, to remove insoluble material, and the supernatant was collected and stored at −80°C. Protein concentration of each sample was determined by the method of Bradford using a protein assay dye reagent (Bio-Rad, Hercules, CA). Samples were dissolved in LDS sample buffer (Invitrogen) and heated at 70°C for 7 min. Equal amounts of the samples (20–30 µg) were electrophoresed in NuPAGE 4–12% Bis-Tris gel (Invitrogen) and transferred to a 0.45- µm nitrocellulose membrane (Bio-Rad Laboratories, Inc.). The membrane was immersed in 5% nonfat milk or BSA, 0.1% Tween 20 and dissolved in Tris-buffered saline to block the nonspecific binding. The membranes were incubated overnight at 4°C with the following primary antibodies: mouse polyclonal anti-HRAS (diluted 1∶1,000; Abnova, CA), mouse monoclonal anti-CDKN2A (diluted 1∶500; Abnova, CA), mouse monoclonal anti-p53 (diluted 1∶500; clone DO-7; BD Transduction Laboratories), mouse monoclonal anti-VEGFA (diluted 1∶500; Santa Cruz Biotechnology, CA), rabbit polyclonal anti-TGFβ1 (diluted 1∶1000; Novus Biologicals, CO) and mouse monoclonal anti-OPN (diluted 1∶500; Santa Cruz Biotechnology, CA). The membranes were then washed and incubated for 90 min. at RT with a secondary antibody that included horseradish peroxidase-conjugated goat anti-rabbit or anti-mouse IgG (diluted 1∶1,500; Santa Cruz Biotechnology). Western blots were normalized using a monoclonal anti-**β-actin antibody (diluted 1∶5,000; Sigma Chemicals, St. Louis, MO). The specific signals were visualized by ECL reagent (GE Healthcare Bio-Sciences, Piscataway, NJ) after exposition to ECL film. The relative density of the polypeptide bands detected on the ECL film was determined using the spot denso tool of the AlphaEaseFC software.

### GEO Dataset computational analysis

Computational analysis was performed to further investigate the differences in the OPN, VEGFA, TGFβ1, FGF2, EGFR, EGF, p14^ARF^, p16^INK4A^, p53, KRAS, HRAS, NRAS, ARAF, BRAF, RAF1, RKIP, MMP2, MMP9, TIMP1, TIMP2 and CyclinD1 transcript levels among different urinary bladder cancer types, its metastatic counterpart and the relative normal tissue. Four publicly available Gene Expression Omnibus (GEO) datasets were analyzed for this reason, with GEO series accession numbers GSE89, GSE7476, GSE3167 and GSE12630 [19], [20], [21]. Expression patterns of the genes were extracted from the normalized datasets and were expressed as mean levels of the log_2_ intensity (**Table S2**).

### Gene ontology (GO) and Kyoto Encyclopedia of Genes and Genomes (KEGG) analysis

Gene ontology (GO) and Kyoto Encyclopedia of Genes and Genomes (KEGG) molecular pathway analysis were performed to identify possible enrichment of genes with specific biological themes (http://www.genome.jp/kegg/pathway.html).

### Statistical analysis

Microarray data were normalized by the global median of the spot intensities. Each gene was tested for its significance in differential expression using a z-test. GOI expression levels were first evaluated by the one-sample Kolmogorov-Smirnov Goodness-of-Fit test, in order to determine whether they followed a normal distribution pattern. The non-parametric Spearman rank correlation was used to examine pair-wise correlations between the mRNA levels and their association with continuous variables (age, smoking, tumor stage/grade). The Mann-Whitney U test was used to examine the expression status of the genes with the various clinicopathological parameters after stratification. The Kaplan-Meier method was used to estimate overall survival as a function of time. Survival differences were assessed by Log-rank (Mantel Cox) and Gehan-Breslow-Wilcoxon tests. Numerical values are expressed as the mean±standard deviation (SD), and medians. Data extracted from the GEO databases were statistically compared by the Mann-Whitney U test. Statistical significance was set at the 95% level (p<0.05). All statistical analyses were performed with SPSS 11.5 (SPSS, Chicago, IL).

## Results

### Immunohistochemical results

**Figure 1:**
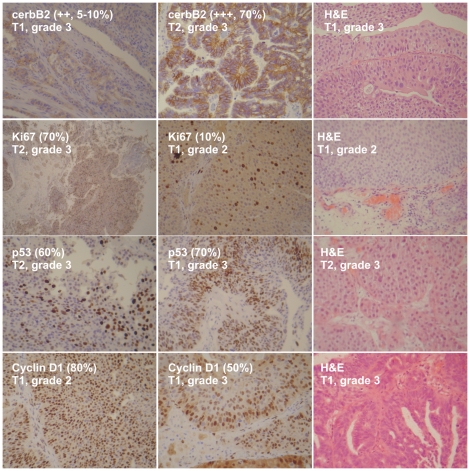
Representative immunohistochemical analysis for tumors of T1/grade 2, T1/grade 3, T2/grade 3. Tumors were stained with anti-cerbB2, anti-Ki67, anti-p53 and anti-Cyclin D1. H&E, Representative hematoxylin-eosin slides.

Tumor samples were stained with antibodies for ErbB2, cyclin D1, p53 and Ki-67 (**Figure 1**). If present, anti-ErbB2 staining in tumor samples is a membrane staining, diffuse in the urothelium. All BC samples (100%) showed moderate/strong (++, +++) immunostaining, whereas no BC sample showed no/weak immunostaining (0, +) for ErbB2. Thresholds for high labeling indices were set for Ki-67 at ≥10% positive tumor nuclei and for p53 at 10 and 20%. T1-grade 1/2 tumors showed weak staining for anti-Ki-67 (7.5% and 40%, respectively), whereas T1/2-grade 3 tumors exhibited the strongest immunostaining (+++, >70%). Similarly, T1-grade 1/2 tumors showed weak staining for anti-p53 (10% and 35%, respectively), whereas T1/2-grade 3 tumors exhibited the strongest immunostaining (53–70%). Regarding Cyclin D1, all tumors showed intense staining for anti-Cyclin D1, whereas those of a higher grade exhibited comparatively lower immunostaining. For T1-grade 1/2 tumors, staining for anti-Cyclin D1 was 80%; whereas for T1/2-grade 3 tumors immunostaining was 50%.

### Microarray expression and clustering on a 22-gene set

**Figure 2:**
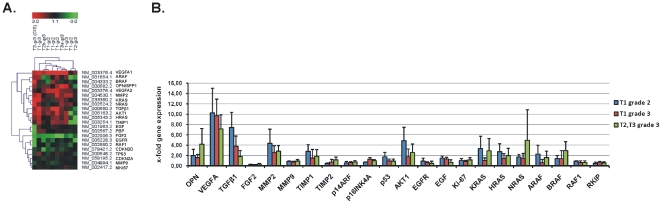
Two-way average-linkage hierarchical clustering with Euclidian distance for the genes of interest (GOIs) in 10 TCC samples *vs*. 5 controls. Each row in the diagram represents a gene and each column a tumor sample. The color saturation represents differences in gene expression across the tumor samples; red indicates expression higher than the median (black), and green indicates expression lower than the median. The color intensity indicates degree of gene regulation (**A**). Fold expression of the GOIs with respect to tumor histology, as detected by microarray analysis (**B**).

We previously identified 831 genes that were differentially expressed in all 10 BC samples, simultaneously. Of these, 33 genes were up-regulated and 85 genes were down-regulated in all bladder cancer samples compared to the 5 normal tissues, simultaneously (data not shown). In the present study, we performed two-way average-linkage hierarchical clustering with Euclidian distance for a 22-gene set that included the following genes: VEGFA, ARAF, BRAF, OPN (SPP1), MMP2, KRAS, NRAS, TGFβ1, AKT1, HRAS, TIMP1, EGF, RKIP (PBP), FGF2, EGFR, RAF1, CDKN2D, TP53, CDKN2A (p14^ARF^/p16^INK4A^), MMP9 and MKI67, in 10 BC samples vs. 5 controls. A detailed view of the sample cluster dendrogram is displayed in **Figure 2A**. We analyzed the log_2_ transformed fold expression pattern of the GOI and partitioned the tumors into 3 main groups based on the differential expression of these 22 genes: The first principle branch contained T1-grade 3, T2-grade 3, T1-grade 2 and T3-grade 3 tumors. The second branch consisted of T1-grade 2 and T2-grade 3 tumors. The third branch consisted of tumors with in situ carcinoma, T2-grade 3 (CIS). The 22-gene set was divided into two main clusters. The first cluster was composed of genes that were over-expressed (VEGFA, ARAF, BRAF, OPN, MMP2, KRAS, NRAS, TGFβ1, AKT1, HRAS and TIMP1) and the second cluster contained genes that were equally or under-expressed (EGF, RKIP/PBP, FGF2, EGFR, RAF1, CDKN2D, TP53, CDKN2A, MMP9 and MKI67) in TCC vs. normal tissue. The relative expression levels of the GOIs in each tumor group were expressed as a ratio of the normalized expression levels from the tumor group to the mean levels of the normal tissues, which was set to 1.0 (**Figure 2B** and **Table S2**).

The fold expression (mean±SD) of the GOIs in each one of the three tumor groups compared to the normal tissue, as acquired by our microarray analysis, is depicted in (**Figure S2**).

### Verification of mRNA and protein expression relative to clinicopathological characteristics

**Figure 3:**
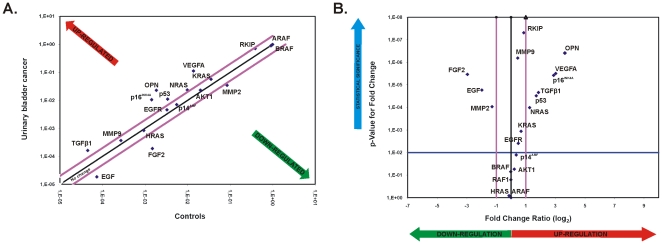
The Scatter Plot graphs the expression level (2^∧-ΔCt^) of each gene in BC samples versus the control samples. The black line indicates fold changes of 1. The pink lines indicate the threshold in gene expression fold-change (2-fold difference). qPCR revealed up- and down-regulated genes in urinary bladder cancer. Total RNA from the normal adjacent tissue and urinary bladder cancer were characterized in technical triplicates, and the relative expression levels for each gene in the two tissue types were plotted against each other in the Scatter Plot. VEGFA, OPN, p14^INK4A^, p6^CDKN2A^, NRAS and TGFβ1 were up-regulated, while FGF2 and EGF were down-regulated by at least two-fold (outside the purple lines). The genes KRAS, MMP2, AKT1, p53, EGFR, MMP9 and HRAS exhibited equal expression between bladder cancer and normal tissue (**A**). The Volcano Plot graphs the log_2_ of the fold change in expression of each gene between the BC samples versus its p-value from the t-test. The black line indicates fold changes of 1. The pink lines indicate the threshold in gene expression fold-change (2-fold difference). The blue line indicates the threshold for the p-value of the t-test (0.01) (**B**).

The mRNA expression of the genes: VEGFA, ARAF, BRAF, OPN (SPP1), MMP2, KRAS, NRAS, TGFβ1, AKT1, HRAS, EGF, RKIP (PBP), FGF2, EGFR, RAF1, TP53, CDKN2A (p14^ARF^/p16^INK4A^), MMP9 was determined by qPCR in both bladder cancer and normal tissue (**Table S3**). Scatter plots were constructed for better visualization of the genes that were up-regulated (>2-fold), down-regulated (<2-fold) or equally expressed (2-fold difference threshold) between BC and controls. Volcano plots graphing the log_2_ of the fold change in the expression of each gene between BC samples vs. its p-value from the t-test, were also constructed (**Figure 3**).

The genes OPN, VEGFA, TGFβ1, p16^INK4A^, p53, RKIP and NRAS were significantly over-expressed in BC vs. normal tissue (p<0.001; t-test) (**Figure S3A**). The mean mRNA expression values between BC and normal tissue for each gene are depicted in **Table 2**.

**Table 2:** The genes OPN, VEGFA, TGFβ1, p16^INK4A^, p53, RKIP and NRAS were significantly over-expressed in BC vs. normal tissue (p<0.001).

Gene name	Mean expression ± SD in BC (median value)	Mean expression ± SD in Normal Tissue (median value)	p value *
OPN (SPP1)	0.0653±0.0820 (0.03115)	0.0112±0.0248 (0.002262)	p<0.0001
VEGFA	0.1644±0.1237 (0.1419)	0.0203±0.0170 (0.01662)	p<0.0001
TGFβ1	0.0003±0.0003 (0.0001815)	0.0001±0.0001 (4.950e-005)	p = 0.0004
p16^INK4A^	0.0424±0.0611 (0.01351)	0.0034±0.0079 (0.001294)	p = 0.0002
p53	0.0156±0.0112 (0.01303)	0.0100±0.0118 (0.003308)	p<0.0001
RKIP	0.7478±0.2009 (0.7648)	0.5178±0.2437 (0.5757)	p = 0.0001
NRAS	0.0555±0.1498 (0.02401)	0.0155±0.0171 (0.01067)	p = 0.0026

*Mann-Whitney U test.

The mRNA expression levels of the genes p14^ARF^, AKT1, HRAS, ARAF, BRAF, RAF1, MMP9, EGFR and KRAS, did not differ significantly between BC and control tissue (p>0.01; t-test). The mean mRNA expression values between BC and normal tissue for each gene, were as follows: p14^ARF^, 0.0325±0.0488 vs. 0.0100±0.0118 (p = 0.1087); AKT1, 0.0321±0.0230 vs. 0.0262±0.0174 (p = 0.3632); HRAS, 0.0015±0.0021 vs. 0.0015±0.0021 (p = 0.9752); ARAF, 0.9931±0.0047 vs. 0.9942±0.0040 (p = 0.5201); BRAF, 0.8933±0.0657 vs. 0.9053±0.0486 (p = 0.8999); RAF1, 0.9348±0.0527 vs. 0.9419±0.0293 (p = 0.6681); MMP9, 0.0014±0.0015 vs. 0.0023±0.0054 (p = 0.1265); EGFR, 0.0078±0.0073 vs. 0.0049±0.0048 (p = 0.0948); KRAS, 0.0876±0.0997 vs. 0.0773±0.0900 (p = 0.4035; Mann-Whitney U test) (**Figure S3B**).

EGF, FGF2 and MMP2 exhibited significant under-expression in BC vs. normal tissue: EGF, 0.00004±0.000047 vs. 0.000151±0.000235 (p = 0.0017); FGF2, 0.0003±0.0004 vs. 0.0023±0.0019 (p<0.0001); MMP2, 0.0752±0.0858 vs. 0.1341±0.0834 (p = 0.0007; Mann-Whitney U test) (**Figure S3C**).

**Figure 4:**
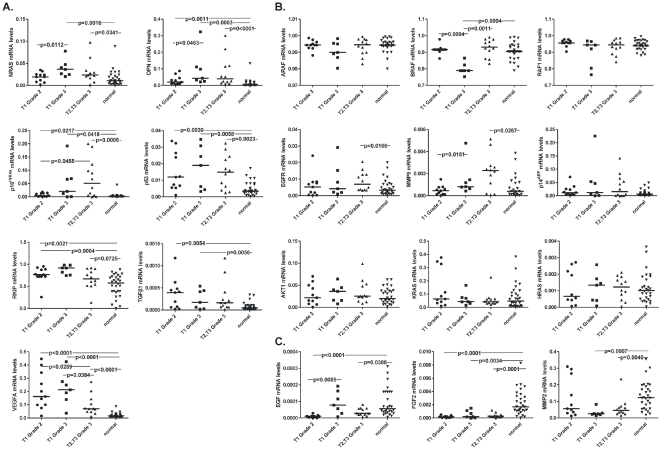
Scatterplot depicting the mRNA levels of the genes that were over-expressed in various urinary bladder cancers of different stage/grade, versus normal tissue. Groups pairs were statistically compared using the Mann-Whitney U test. Bars depict the median values (**A**). Scatterplot depicting the mRNA levels of the genes that were equally expressed among urinary bladder cancers of various stage/grade, and normal tissue. Groups pairs were statistically compared using the Mann-Whitney U test. Bars depict the median values (**B**). Scatterplot depicting the mRNA levels of the genes that were under-expressed in various urinary bladder cancers of different stage/grade, versus normal tissue. Group pairs were statistically compared using the Mann-Whitney U test. Bars depict the median values (**C**).

The mRNA expression of the genes that exhibited over-, equal or under-expression according to our qPCR analysis was also investigated relative to the stage/grade of the tumors. All statistically significant differences in expression of each gene among the tumor groups T2/T3-grade 3, T1-grade 3 and T1-grade 2, are depicted in **Figure 4**.

**Figure 5:**
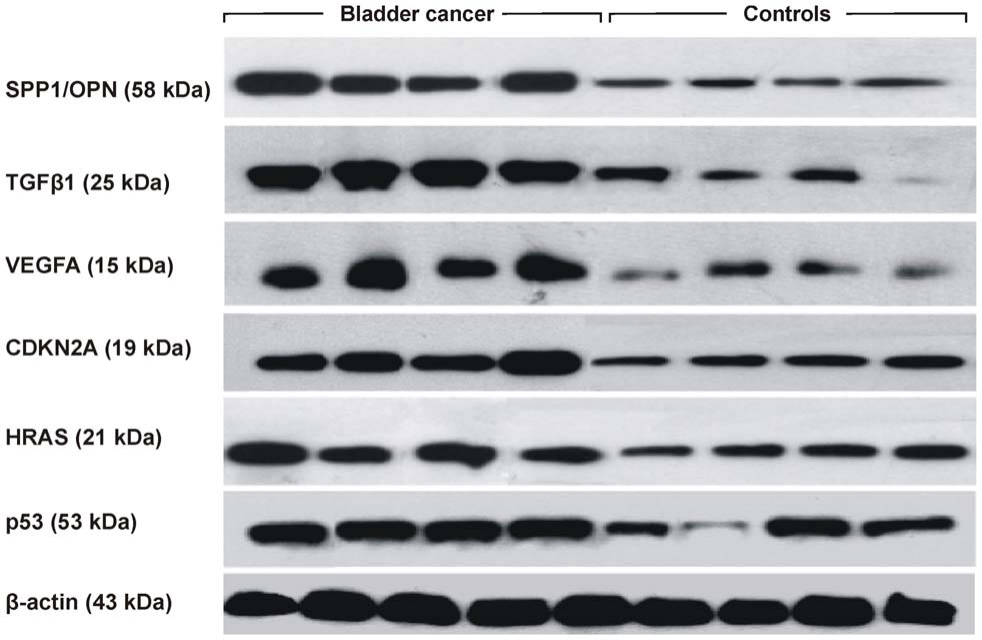
Representative bands depicting the expression of SPP1/OPN (58 kDa), TGFβ1 (25 kDa), VEGFA (15 kDa), CDKN2A (19 kDa), HRAS (21 kDa) and p53 (53 kDa) proteins in 4 BC and 4 control samples, using Western blotting. β-actin (43 kDa) was used as loading control.

The expression of OPN (SPP1), TGFβ1, VEGFA, CDKN2A (p14^ARF^/p16^INK4A^), HRAS and TP53, was also verified at the protein level, by densitometric analysis of the Western blots (**Figure 5**). The normalized protein levels in BC vs. normal tissue were as follows: OPN, 0.645±0.287 vs. 0.261±0.020 (p = 0.0286); TGFβ1, 0.837±0.114 vs. 0.300±0.195 (p = 0.0286); VEGFA, 0.678±0.183 vs. 0.295±0.133 (p = 0.05); CDKN2A, 0.687±0.157 vs. 0.429±0.088 (p = 0.0286); HRAS, 0.663±0.258 vs. 0.420±0.121 (p = 0.1143); p53, 0.760±0.167 vs. 0.448±0.295 (p = 0.2000; Mann-Whitney U test).

### Correlation between mRNA/protein expression of the GOIs and survival rates of the bladder cancer patients

A total of 30 urinary bladder cancer cases were investigated for overall survival rates. Patients with tumors of stage 1 presented better overall survival compared to those of stage 2 or 3 [p = 0.0008, χ^2^ = 14.32, Log-rank (Mantel Cox) test; p = 0.0041, χ^2^ = 8.260, Gehan-Breslow-Wilcoxon test]. Moreover, patients with grade 2 tumors showed a better overall survival vs. those of grade 3 tumors [p = 0.0475, χ^2^ = 6.094, Log-rank (Mantel Cox) test].

The median value for MMP2 mRNA expression in the bladder cancer samples was 0.038. The cases were divided into two groups with expression above and below this median value. Similarly, we divided the cases into groups based on the median values for MMP9 (0.0007), OPN (0.031), VEGFA (0.141), TGFβ1 (0.0001), FGF2 (0.0002), p14^ARF^ (0.011), p16^INK4A^ (0.013), p53 (0.013), AKT1 (0.025), EGFR (0.005), EGF (0.00002), HRAS (0.0011), KRAS (0.051), NRAS (0.024), ARAF (0.994), BRAF (0.916), RAF1 (0.956), RKIP (0.764) mRNA expression (**Table 2**).

**Figure 6:**
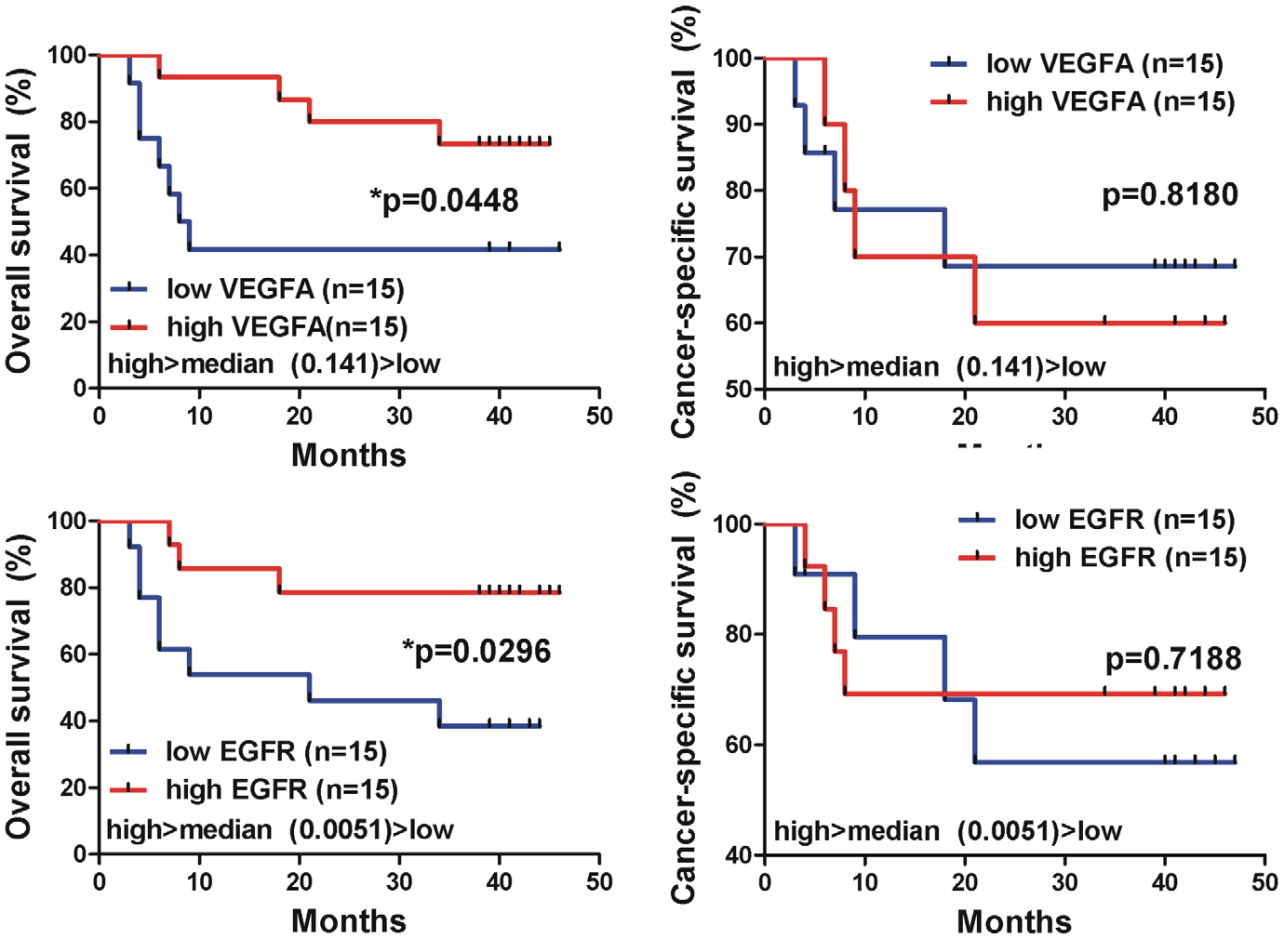
Kaplan-Meier curves depicting overall survival (%) of the urinary bladder cancer patients, regarding the mRNA expression of the GOIs. The cases whose tumors exhibited low levels of VEGFA (<median value, 0.141) and EGFR (<median value, 0.0051) mRNA expression, exhibited worse overall survival rates, than the cases expressing increased VEGFA (>median value, 0.141) and EGFR (>median value, 0.0051) mRNA levels [for VEGFA, p = 0.04; for EGFR, p = 0.02; Log-rank (Mantel Cox) test]. Differences in survival were assessed using Log-rank (Mantel Cox) test. Statistical significance was set at the 95% level (p<0.05).

The cases whose tumors exhibited low levels of VEGFA (<median value, 0.141) and EGFR (<median value, 0.0051) mRNA expression exhibited worse overall survival rates than the cases expressing increased VEGFA (>median value, 0.141) and EGFR (>median value, 0.0051) mRNA levels, respectively [for VEGFA, p = 0.04; for EGFR, p = 0.02; Log-rank (Mantel Cox) test]. Patients with high TGFβ1, FGF2, p14^ARF^, AKT1, RKIP, KRAS and p53 mRNA levels also showed a better overall survival pattern, however the correlation was not statistically significant. Kaplan-Meier curves for all the GOIs are depicted in **Figure 6**.

### Comparison of GEO Datasets

We further compared our qPCR results to 4 publicly available BC microarray GEO datasets: GSE89 by Dyrskjot et al. (2003) [19]; GSE7476 by Mengual et al. (2009) [21]; GSE3167 by Dyrskjot et al. (2004) [20] and GSE12630 by Monzon et al. (2009) [22]. All mean log_2_ transformed ratios of BC samples vs. controls are depicted in **Table S2**.

Dataset GSE12630 contained data only from transitional cell carcinoma of urinary bladder and metastatic urothelial carcinoma. Since no data from the normal tissue were available for this dataset, we extracted normal tissue data from dataset GSE89, and computed the mean log_2_ transformed ratios of BC vs. these extracted data, which we used as controls (**Table S2**).

### Correlation analysis

We explored the correlation of the mRNA levels of the GOIs in BC as well as in normal tissue, performing the Spearman rank test (**Table S4**). In BC, MMP2 was significantly correlated with BRAF (p = 0.032), RAF1 (p = 0.039) and RKIP (p = 0.014). MMP9 correlated with VEGFA (p = 0.005) and FGF2 (p = 0.016). OPN correlated with NRAS (p = 0.027). VEGFA correlated with FGF2 (p = 0.019). TGFβ1 correlated with p53 (p = 0.033), EGFR (p = 0.048) and RKIP (p = 0.032). FGF2 correlated with EGFR (p = 0.015), HRAS (p = 0.005) and KRAS (p = 0.029). p14^ARF^ correlated with p16^INK4A^ (p<0.0001), KRAS (p = 0.016), NRAS (p = 0.009) and ARAF (p = 0.044). p16^INK4A^ correlated with EGF (p = 0.017) and NRAS (p = 0.002). p53 correlated with AKT1 (p<0.0001), EGFR (p = 0.002) and RAF1 (p = 0.041). AKT1 correlated with EGFR (p<0.0001) and RKIP (p = 0.022). EGF correlated with NRAS (p = 0.027). HRAS correlated with NRAS (p = 0.007) and ARAF (p = 0.030). KRAS correlated with RKIP (p = 0.035). NRAS correlated with ARAF (p = 0.003) and BRAF (p = 0.004). ARAF correlated with BRAF (p = 0.001) and RAF1 (p = 0.001). BRAF correlated with RAF1 (p = 0.040). All positive and negative interactions among the genes, indicating their synergism in bladder tumorigenesis, can be deduced from the correlation coefficient values depicted in **Table S4**.

Moreover, we used the BioNetwork Tools from www.pubgene.org, in order to investigate the relationships between the GOIs. HRAS confirmed its role as a key molecule, since it presented interaction with the majority of the GOIs (nineteen interactions). OPN, TP53, BRAF and EGFR exhibited the second highest number of significant interactions (18 of the GOIs), whereas VEGFA, RAF1, CDKN2A. ki67, ERBB2 and FGF2 were associated with 17 of the GOIs. NRAS was associated with 16 GOIs, KRAS, MMP2 and MMP9 with 15 GOI, TGFβ1 with 14 GOIs, ARAF with 13 GOIs, EGF with 6 GOIs and RKIP/PEBP1 with 5 of the GOIs, according to the literature (**Figure S4**).

### Hierarchical Clustering (HCL) of the Selected Genes

We performed hierarchical cluster analysis on this selected small gene dataset. HCL analysis classified the tumors based on their degree of aggressiveness, as expected (**Figure S2** and **Figure S5–A**). HCL was performed on the microarray expression values, where the average of all samples in a tumor category was calculated and further processed.

### Principal Component and Correspondence Analysis (PCA and CA)

We also performed PCA and CA analysis on the selected dataset as it was obtained from microarray analysis. Clusters from HCL were made public in order to better visualize genes in those analyses. The results are presented in **Figure S5B–D**. Also, when samples were analyzed for their average expression values for the selected dataset, they were classified according to their clinical phenotype i.e. T1-grade 3 and T2/T3-grade 3 were grouped together while T1-grade 2 was set in a more distant location of the 3-scale axis.

### Gene Ontology (GO) and Kyoto Encyclopedia of Genes and Genomes (KEGG) Analysis

**Figure 7:**
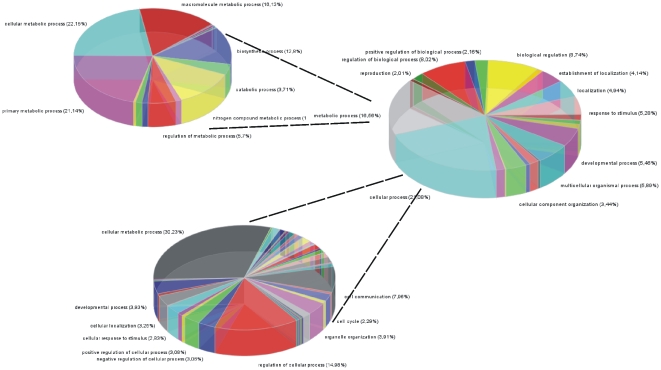
GO analysis of the selected genes revealed functions related to cell death, cell proliferation, metabolism and signal transduction.

GO and KEGG molecular pathway annotation analysis in the selected genes is presented in **Figures 7** **,** **8** and **Table S5**, respectively. Interestingly, KEGG analysis of our selected dataset, revealed that the most significant pathway was that of Bladder Cancer (p = 1.5×10^-31^).

**Figure 8:**
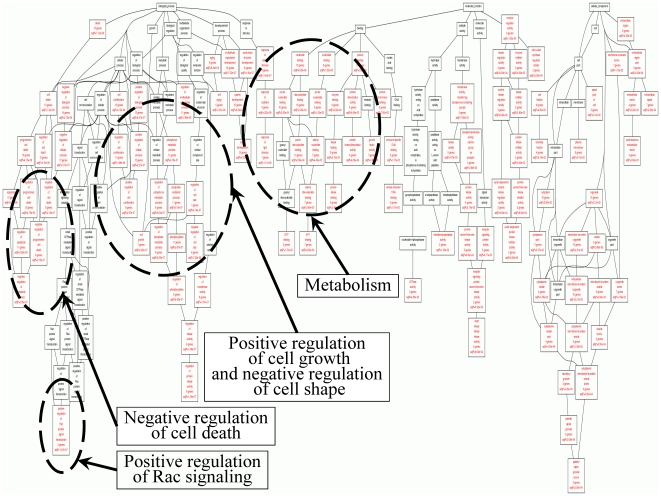
Further resolution of GO functions showed that the majority of genes (∼30%) are related to metabolism. However, despite the small size of the dataset that we used, it appeared that those selected genes participate significantly in a variety of cellular functions.

## Discussion

As additional molecular determinants are added to a constantly growing list of prognostic indicators for BC, there is a growing need to combine such markers into logical groups and employ them for determination of outcome and therapeutic response in individual patients. It is clear that several molecular alterations can operate together to influence the pathogenesis of BC and its ultimate clinical behavior. Analyzing these alterations in combination may therefore provide deeper insight into the pathobiology of the disease, while also generating panels of markers that may be able to better predict patient outcome and treatment response.

In the present study, we analyzed the expression profile of genes involved in cell cycle control, DNA damage repair, apoptosis, signal transduction, transcription factors, angiogenesis, cellular proliferation, invasion and metastasis. Our results showed an observable overlap between the tumor and normal urothelium samples with respect to the expression of the GOIs. Nevertheless, the detected changes in the expression of the GOIs were statistically significant in the tumor vs. paired non-tumor samples. The aforementioned overlap in the expression of the GOIs can be explained by the fact that overt carcinomas are frequently surrounded by histologically normal, but genetically aberrant urothelium. Increasing evidence suggests that TCCs and the surrounding urothelium share common genetic and epigenetic alterations. This genetic similarity may be the result either of the field cancerization effect or of intraepithelial migration. The two mechanisms, together with intraluminal seeding, appear to be strongly responsible for multi-focal disease as well as for localized and distant recurrences within the urinary bladder.

### Cell Cycle Control & DNA Damage Repair genes

Different studies have indicated that alteration in cell-cycle regulation is a key event in determining the biological behavior of bladder cancer [23]. P53 controls the cell cycle and is also involved in angiogenesis, apoptosis and DNA repair [24]. Most previous studies have demonstrated that the rate of p53 expression in patients with high-grade tumors is higher than in patients with low-grade tumors [25], [26], [27]. However, Halimi et al. and Moch et al. showed a similar difference between BCs of low- and high-grade [28], [29]. In accordance with these data, we also detected higher p53 mRNA and protein levels by qPCR and Western blotting in BC vs. normal tissue (p<0.0001). However, qPCR did not reveal differences between high and low-grade tumors. The fold differential expression rate also did not differ significantly among tumors of low or high grade, as acquired by our microarray analysis. Immunohistochemistry on the other hand, showed higher p53 staining for grade 3 tumors vs. those of grade 2.

The most common genetic damage in BC is partial loss of chromosome 9. The area surrounding 9p21 where the CDKN2A/ARF gene is located is one of the major sites for deletion. p16^INK4a^ over-expression has been previously detected in urothelial carcinomas [30], [31], [32], [33]. Moreover, the expression of p16^INK4A^ has been statistically associated with disease stage but not with either tumor grade or disease progression [31]. p16^INK4A^ expression has also been related to recurrence and survival [34]. In the present study, the mRNA levels of p16^INK4A^ were higher in BC vs. normal tissue, as assessed by qPCR. Western blot analysis verified the higher protein expression for CDKN2A in BC vs. normal tissue. Tumors of T2/T3-grade 3 exhibited higher p16^INK4A^ mRNA levels vs. those of lower stage/grade. Finally, we could not relate p16^INK4A^ expression to survival, probably due to the small sample number. p14^ARF^ did not show any significant differences between BC and normal tissue, or among tumors of different histology. In contrast, high p14^ARF^ expression showed a tendency to correlate with favorable prognosis.

Cyclin D1 plays an important role in the cell cycle, binds to cyclin-dependent kinases (CDK4/6), and promotes phosphorylation of RB1, orchestrating progression through the G1 restriction point. Recently, Del Rey et al. found that CCND1 amplification in homogeneously staining regions was present in 6 out of 14 CIN-positive tumors; 3 of them also showed amplification of this gene in double minutes [35]. Moreover, Levidou et al. used IHC and showed that Cyclin D1 expression decreased with increasing grade, tumor T-category and in muscle-invasive carcinomas [36]. In the present study, we also performed IHC in order to investigate the expression pattern of Cyclin D1 in BC. Our data showed intense staining for anti-Cyclin D1 in all tumors, corroborating that the gene CCND1 is frequently amplified in bladder cancer. Notably, in accordance with the results of Levidou et al. [36], our data found that T1-grade 1/2 tumors exhibited more intense immunostaining compared to T1/2-grade 3 tumors, suggesting that Cyclin D1 over-expression is more important during early stages of bladder carcinogenesis.

### Apoptosis

Alterations in apoptotic pathways contribute to tumorigenesis and progression, as they allow cancerous cells to survive longer, resist normally harmful stresses, and become more invasive [37]. In the present study we paid specific attention to changes in osteopontin (OPN, SPP1) expression, due to its multiple biological functions. The constitutive expression of OPN has been reported to be involved in the process of tumor carcinogenesis and metastasis of multiple human malignancies [38].

Recently, two research groups used immunohistochemistry and found that OPN was over-expressed in bladder cancer [39], [40]. We performed microarray analysis and classified OPN into the genes that exhibited >2-fold differential expression in BC vs. normal tissue. This observation was also verified by qPCR (p<0.0001) and Western blot experiments (p = 0.0286). One observation was that grade 3 tumors presented higher OPN mRNA levels vs. those of grade 2. However, OPN expression did not seem to be significantly correlated with tumor stage or grade. Ke et al. (2009) also reported lack of correlation with recurrence-free survival, but high OPN expression was a significant predictor for cancer-specific survival [40]. Our data did not show a significant correlation of OPN expression and patient survival. Moreover, OPN score and stage have been previously found to be significantly correlated with bladder cancer location [39], [40]. Osteopontin may also play a role in suppressing tumor growth in vivo. Morimoto et al. (2002) identified the OPN gene as a TP53 target gene and found that its expression was up-regulated by DNA damage-induced TP53 activity and by adenovirus-mediated transfer of the human TP53 gene [41]. They demonstrated that the OPN gene has a functional TP53-responsive element in its promoter region and confirmed an interaction between the OPN promoter and TP53 protein in vivo. The results suggested that OPN is a direct transcriptional target of TP53. In accordance with this, our Pubgene analysis verified an association between OPN and TP53. Further evidence supporting the interaction between OPN and TP53, was derived from our data retrieved from HCL and PCA analyses for genes, as well as CA analysis for our microarray dataset.

### Signal Transduction Molecules and Transcription Factors

Signal transduction involves diverse pathways that modulate growth signals from cell-surface receptors to transcription factors in the nucleus. Alterations within these pathways can result in the deregulation of genes that control cellular homeostasis. One of the most important signaling pathways in BC is the Ras-mitogen-activated protein kinase (MAPK) signaling cascade.

When RAS family members become activated, through physiological stimuli or through mutation [7], [17], [42], [43], they signal to key pathways involved in cell growth, survival, differentiation and migration, including the RAF-MAPK [44], PI3K [45], and the RALGEF (RALGDS; ral guanine-nucleotide-dissociation stimulator) [46] pathways. In non-muscle invasive BC, HRAS has been shown to be the most commonly mutated RAS family oncogene [7], [47], [48]. Several point mutations have been identified in the HRAS oncogene in BC, with the most frequent mutations occurring at codons 12, 13 and 61 [7], [49], [50], causing an inability to hydrolyze GTP to GDP [51]. This mutation is responsible for constitutive activation of the HRAS protein and the ability to transform NIH 3T3 cells. Moreover, a high percentage of BC samples (77%) has been previously reported to exhibit over-expression in at least one RAS family gene compared to adjacent normal tissue [7]. KRAS and NRAS genes were previously reported to show the highest levels of over-expression in BC vs. normal tissue [7]. In the present study, NRAS was >2-times over-expressed in BC vs. controls (p = 0.0026), whereas both KRAS and HRAS exhibited equal mRNA and protein levels between the 2 tissues. Finally, RAS expression levels did not correlate with patient survival.

Another oncogene implicated in BC is the epidermal growth factor receptor (EGFR). It's over-expression has been described in several tumors including bladder [52], [53]. Pathological expression of EGFR leads to uncontrolled cell proliferation. It also results in increased angiogenesis and reduced apoptosis, processes necessary for continued malignant growth. Preclinical studies in bladder cancer cell lines confirm that EGFR inhibitors can inhibit the growth of urothelial cancer cells in vitro [54]. In addition, there is a correlation between high EGFR expression and adverse clinical and pathologic characteristics including poor outcome, advanced stage and high grade [55]. In bladder cancer, the over-expression of EGFR has been widely reported [55], [56], [57], [58], [59] and several studies have shown EGFR positivity to be associated with high tumor stage, tumor progression, and poor clinical outcome [55], [57]. In accordance with this, our data showed moderate immunostaining for T1-grade 3 and strong immunostaining for T2-grade 3 tumors, whereas no BC sample showed no/weak immunostaining. Furthermore, our qPCR analysis revealed a significant difference in the expression of EGFR, only between T2/T3-grade 3 and normal tissue (p = 0.0166). Low EGFR mRNA levels were associated with poor prognosis (p = 0.0296), contrasting previous reports [60].

AKT1 is one of three family members of the serine-threonine kinases that are activated by phosphorylation of Thr308 by PDK1 and Ser473 by TORC2 [61], [62]. AKT occupies a key regulatory node in the PI3K pathway, below which the pathway branches significantly to influence a wide range of cellular processes that promote cell cycle progression, cell growth, energy metabolism and resistance to apoptosis. Apart from mutations activating AKT1 in several cancers, including bladder [63], [64], there are limited data regarding its expression in urinary bladder cancer. In the present study, AKT1 showed >2-fold expression in BC using microarrays, however, this was not verified by qPCR, where we detected equal mRNA levels between BC and normal tissue. Moreover we did not detect a significant change between tumors of low vs. high grade. Also, AKT1 mRNA expression levels did not correlate with patient overall survival.

The RAF (ARAF, BRAF and RAF1) and MEK (MEK1/MAP2K1 and MEK2/MAP2K2) family of proteins are critical downstream mediators of the RAS and other oncogenic signaling pathways [8], [65], [66], [67]. The RAF family of proteins are responsible for phosphorylating MEK, which results in its activation [68]. Involvement of BRAF mutations in the development of transitional cell carcinoma of the bladder has been reported to be rather infrequent [67]. RKIP, also known as phosphatidylethanolamine-binding protein 1 (PEBP-1) or prostatic binding protein (PBP), was initially characterized to be involved in many different physiologic activities, including reproduction and neurophysiology [69]. Previous findings, however, have identified RKIP as a modulator of apoptosis and metastasis through regulation of important signaling cascades, i.e., the RAF-MEK-ERK kinase cascade, G protein-coupled receptors, and the NF-κβ pathway [70], [71]. RKIP expression has been reported to be diminished in many tumors and completely absent in metastases [72], [73], [74]. We recently reported a significant reduction in RKIP expression in BC vs. normal tissue, followed by elevated levels of BRAF gene expression [6]. In the present study, low RKIP expression in BC was verified only by microarray analysis. Our PCR analysis revealed high expression for RKIP (p = 0.001) and equal expression for BRAF between BC and normal tissue, perplexing thus the elucidation of its expression profile in BC. Notably, high RKIP expression levels showed a trend for favorable overall patient survival, whereas high BRAF expression levels tended to be more frequent in patients with worse overall survival. However, the correlations were not statistically significant. We have previously reported positive correlations among all 3 RAF genes and negative correlation between the expression of BRAF and RKIP in BC [6]. The present study corroborates our previous results, both by pubgene analysis and Spearman's bivariate correlation-coefficient test.

### Angiogenesis

Angiogenesis is essential for tissue development, wound healing and reproduction [75] and is an indispensable requirement for tumor progression, invasiveness and metastasis [76].

Of the angiogenic factors, VEGF has been identified as a crucial regulator of normal and pathological angiogenesis. VEGF produces a number of important biological effects such as endothelial mitogenesis and migration, extracellular matrix remodeling via induction of proteinases, increased vascular permeability and maintenance of newly formed vasculature [77]. There is substantial evidence to suggest that the essential role of VEGF is to act as a prognostic marker for the aggressiveness of TCC. Bladder tumors are characterized by markedly increased angiogenesis when compared with the normal urothelium from which they are derived. Thus, VEGF is a crucial growth factor mediating tumor angiogenesis, and its expression has been associated with advanced grade, stage, and recurrence of TCC [78], [79], [80]. VEGF protein has been found to be significantly over-expressed in all grades and stages of TCC [81], [82], [83]. Furthermore, VEGF over-expression has been found to be correlated with the grade, stage, and recurrence of tumors indicating that VEGF-positive tumors are biologically aggressive [81]. Innoue et al. reported that the expression of VEGF in biopsy specimens was correlated with the prognosis of patients with advanced bladder cancer undergoing neoadjuvant chemotherapy and cystectomy [79]. In the present study, VEGFA expression was significantly higher in BC vs. normal tissue, both by microarray analysis (∼10-fold over-expression), qPCR (p<0.0001), and Western blotting (p = 0.05). VEGFA over-expression has been found to be a good indicator of poor survival in patients with TCC [78], [79]. Interestingly enough, our data suggest that high mRNA levels of VEGFA are correlated with favorable overall patient survival (p = 0.0448). Such contradictory results could probably be attributed to the relatively small sample number used for the survival test.

At the early stage of tumor growth, FGF2 expression plays an important role in the regulation of angiogenesis, tumorigenicity and subsequent metastases of human bladder cancer [84]. It has been hypothesized that during wound healing and tumor development the action of heparan sulfate degrading enzymes activates FGF2, thus mediating the formation of new blood vessels. FGF2 may behave as a transforming/oncogenic factor inducing cell proliferation and motility. The interaction with specific receptors leads to unchecked proliferation via the RAS-MAPK pathway. The most common oncogenes and some of the tumor-suppressor genes relevant to BC are components of this pathway [85]. FGF2 protein levels have been reported to be significantly increased in BC tissues [82], [83], [86], [87], [88], and its expression is positively correlated with tumor grade [87]. FGF2 was also reported to be a predictor for recurrence when residual disease was present in the cystectomy specimen [79]. On the contrary, our data suggest a significant down-regulation of FGF2 mRNA levels in BC vs. normal tissue, detected both by microarray (∼0.3-fold) and qPCR (p<0.0001) analysis. This could be attributed to possible experimental error or to the relatively small sample number. We also detected a negative correlation between VEGFA and FGF2 RNA levels (p = 0.019) in BC.

TGFβ1 is regarded as a negative growth regulator because of its potent anti-proliferative effects on many cell types, such as endothelial and epithelial cells and various cell types of haematopoietic origin in vitro [89]. In contrast, over-expression of TGFβ1 has been described in several tumors in vivo [18], [90], [91], [92] and has also been associated with tumor progression and metastasis [93], [94]. In the tumorigenesis of urinary bladder carcinoma, TGFβ1 is also considered a crucial molecule [95]. Recently, Helmy et al. used immunocytochemistry to show that TGFβ1 is significantly increased in BC vs. normal tissue (p<0.01) [96]. Several groups have also found similar results by protein and RNA expression studies [80], [97], [98], [99]. On the other hand, Eder et al. found reduced TGFβ1 mRNA expression in BC vs. normal tissue and elevated TGFβ1 protein levels in superficial forms of BC (Ta-T1) and moderate levels in invasive BCs of stages T2-T3, compared with normal urothelium [99]. We also detected a significant over-expression of TGFβ1 both by microarray and qPCR analysis (p<0.0004), and our data were further validated by western-blotting (p = 0.0286). Moreover, elevated TGFβ1 mRNA levels were noted in BCs irrespective of grade or stage, in good agreement with previous reports [100]. Overall, these data suggest that TGFβ1 protein could be used as an attractive target for anticancer therapy.

Despite the reported over-expression of EGFR in bladder cancer, leading to uncontrolled cell proliferation, increased angiogenesis and reduced apoptosis, to our knowledge, this is the first report regarding the expression of EGF in urinary bladder cancer. Our data imply significantly low levels of EGF, both by microarray and qPCR analysis (p = 0.0017).

Angiogenesis-related molecular markers are commonly altered in urothelial carcinoma of the bladder, making them a target for therapy. Understanding the mechanisms of invasion, metastasis and angiogenesis in BC sets the stage for the development of novel targeted agents.

### Cellular proliferation, Invasion and Metastasis

The gene MKI67 encodes Ki-67, a nuclear protein that is associated with and may be necessary for cellular proliferation. In cancer cells, Ki-67 plays an important role as an index for the replication and the prognosis and is well associated with tumor grade, stage and recurrence [101]. In the present study, T1-grade 1/2 tumors showed weak staining for anti-Ki-67, whereas T1/2-grade 3 tumors exhibited the strongest immunostaining. Our microarray data for MKI67 did not show a >2-fold difference in its expression levels, however T2/T3-grade 3 tumors also showed higher MKI67 expression vs. T1-grade 2 and T1-grade 3 tumors. These results are in agreement with previous studies [102], [103] which showed that Ki-67-positive immunostaining was correlated with tumor grade and muscle invasion.

The matrix metalloproteinases (MMPs) are a family of proteolytic enzymes present in both normal and pathological tissues in which matrix remodelling is involved, including embryonic development, wound healing, arthritis, angiogenesis, tumor invasion and metastasis [104], [105]. The MMPs degrade the components of the extracellular matrix, with MMP1 degrading fibrillar collagen and the gelatinases (MMP2 and MMP9) being important in degrading the basement membrane [106], [107], [108]. MMPs play a role in the invasion of normal tissues by tumors and their subsequent metastatic spread.

Most studies have previously determined MMP2 and MMP9 activity levels in urine or tissue from patients with BC, using gelatin zymography or ELISA [109]. Margulies et al. [110] reported that MMP2 was elevated in the urine of patients with BC, and Ozdemir et al. [111] and Hanemaaijer et al. [112] reported the same for MMP9. More recently, Gerhards et al. [113] found significantly increased MMP2 and MMP9 excretions in patients with bladder cancer, depending on tumor stage and grade. In one of the few studies measuring MMP9 and MMP2 by ELISA in urine, Sier et al. [114] revealed an increased level of both markers in the malignant group compared with a control group that did not include patients with benign urologic disease. Choi et al. found that MMP2 and MMP9 were significantly up-regulated in recurrent BC cases [80]. In the present study, we explored MMP2 and MMP9 activity, measuring their mRNA expression levels by microarray analysis. Our data suggest MMP2 up-regulation in BC vs. normal tissue. However, qPCR did not validate this up-regulation. On the contrary, we noticed MMP2 down-regulation (p = 0.0007) in BC vs. normal tissue. Moreover, in accordance with Gerhards et al. [113], our data show that T2/T3-grade 3 tumors exhibited significantly higher MMP9 levels compared to the control tissue (p = 0.0267). Similarly, MMP9 levels were higher in T1-grade 3 vs. T1-grade 2 tumors (p = 0.0151). Furthermore, MMP9 was correlated both with VEGFA and FGF2 in BC, whereas it has been previously reported to be induced by EGF [115]. This marked increase in MMPs in the malignant group was expected since MMP2 is synthesized by tumor cells or quite commonly by host response to tumor as in fibroblasts, macrophages, and vascular endothelial cells. However, MMP9 was found to be strongly expressed in intravascular and tissue-infiltrating leucocytes [111], and their implication in the pathogenesis of BC has been documented [114].

Apart from MMPs, TIMP expression has also been previously reported in BC [116]. Eissa et al. were the first to use TIMP2, MMP2/TIMP2 ratio, and MMP9/TIMP2 ratio as novel urine markers for BC [117]. Our microarray analysis also showed that TIMP1 was >2-fold up-regulated in T1-grade 2 tumors (2.80±1.29), whereas its expression was <2-fold in T1-grade 3 and T2/T3-grade 3 tumors vs. normal tissue. On the contrary, TIMP2 exhibited low expression levels. Its fold expression in T1-grade 2 tumors was 0.44±0.14, whereas in T1-grade 3 and T2/T3-grade 3 tumors, it was 0.74±0.52 and 1.14±0.56, respectively. Low TIMP2 expression levels in BC vs. benign and control tissues have been previously reported [118], [119], indicating that TIMP2 down-regulation with abundant MMP activation, represents a mechanism of tumor invasion and could be used as a novel prognostic indicator in BC.

### Comparison of GEO Datasets

Regarding Dataset GSE89, TGFβ1, p16^INK4A^, p53, AKT1, HRAS, Cyclin D1, RAF1 and RKIP, exhibited significant over-expression in BC vs. normal tissue. OPN (SPP1), VEGFA, p14^ARF^ and BRAF, exhibited equal expression levels between BC and normal tissue, whereas MMP2, MMP9, TIMP1, TIMP2, FGF2, EGFR, EGF, Ki-67, KRAS, NRAS, and ARAF showed significantly lower expression levels in BC vs. normal tissue.

As for Dataset GSE7476, the genes that exhibited significant over-expression in BC vs. normal tissue were the following: VEGFA, p16^INK4A^, p53, EGFR, EGF, Ki-67, KRAS, NRAS, and cyclin D1. The genes, MMP9, OPN (SPP1), TIMP1, TIMP2, TGFβ1, p14^ARF^, AKT1, HRAS, BRAF, and RKIP, presented equal expression levels between BC and normal tissue; whereas genes that presented significantly lower expression levels in BC vs. normal tissue were MMP2, FGF2, ARAF and RAF1.

Regarding Dataset GSE3167, OPN (SPP1), VEGFA, p53, AKT1, KRAS, HRAS, cyclin D1, RAF1, and RKIP exhibited significant over-expression in BC vs. normal tissue. The genes, MMP2, TIMP1, TIMP2, TGFβ1, Ki-67, NRAS, and ARAF exhibited identical expression between BC and normal tissue; whereas MMP9, FGF2, p14^ARF^, p16^INK4A^, EGFR, EGF and BRAF showed significantly lower expression in BC vs. normal tissue (**Table S2**).

Expression of the majority of the genes demonstrated good agreement among all GEO Datasets. Small differences detected among the 5 GEO Datasets were expected to occur due to divergence in the methodology used in the experimental procedures and in the analysis of each gene. However, log_2_ transformation of the fold-expression rates minimized these differences and provided us with a more definite understanding of the expression profile of the GOIs in BC.

### Gene ontology (GO) and Kyoto Encyclopedia of Genes and Genomes (KEGG) analysis

Furthermore, we performed GO and KEGG molecular pathway annotation analysis in the selected genes. Since the dataset was small, we expected a small number of significant functions to be represented in gene ontology. To our surprise, GO analysis manifested a large variety of functions for the GOIs. The variety of gene functions that were revealed implies the multi-facet character of the selected genes. It appears that they participate in a variety of mechanisms including cell proliferation, cell death (particularly negative regulation of cell death), metabolism and very importantly these same genes participate in cell shape and cytoskeletal re-organization.

Importantly, when we performed an analysis for KEGG pathway participation of our selected dataset, the most significant pathway appeared to be the Bladder Cancer Pathway (p = 1.5×10^−31^). When the certain group of genes was isolated for further processing from microarray experimentation, tumor types were successfully classified based only on these specific genes. This implies that the genes on which our interest was focused for further study, probably characterize bladder neoplasias. Looking further into the functions of the selected genes it appears that, in their majority, they participate in metabolic processes. This was on one hand expected, since tumors are known to be very active metabolically, and on the other hand it was interesting since it confirms the hypotheses stated in recent years that metabolic pathways could be used as therapeutic targets. The participation in many functions of the GOIs in the present study makes them good therapeutic targets. Since these specific genes appear to have key cellular functions and characterize tumor type and grade, future investigations as far as their potency as therapeutic targets are warranted.

Bladder cancer is a disease involving distinct and multiple molecular pathologies. Several of these alterations have been characterized, and yet many more are being discovered. Moreover, great effort has been given towards the possibility of determining subtypes of urological tumors, which in turn would benefit diagnosis, therapy and prognosis. The present work adds to the current knowledge of molecular signature identification of BC. Genes with at least 2-fold differential expression in BC vs. normal tissue, as well as in non-muscle invasive vs. muscle invasive tumors and in low vs. high grade tumors, were identified and ranked. We paid specific attention to alterations in OPN expression, due to its multiple biological functions. GO analysis revealed the multi-functional character of the GOIs, since they participate in a variety of processes, including cell proliferation, cell death, metabolism, cell shape, and cytoskeletal re-organization. Furthermore, KEGG analysis identified the Bladder Cancer pathway as the most significant pathway, in which the selected GOIs participate, providing support for the accuracy of our analysis. Therefore, further research should be undertaken in order to gain more insight into the disease molecular mechanisms underlying the biology of BC which may aid in the administration of more efficacious treatments in clinical practice.

## Supporting Information

Figure S1 Upper panel: Melting curves used for product specificity of the genes MMP2, MMP9, OPN, VEGFA, TGFβ1, FGF2, p14^ARF^, p16^INK4A^, p53, AKT1, EGFR and EGF. NTC, non-template control. Lower panel: Representative examples of PCR products after analysis by electrophoresis on 2% agarose gel.
(TIF)

Figure S2 Differential fold expression of the genes of interest in 10 BC samples vs. 5 controls, as detected by microarray analysis. The Mann-Whitney test was performed to examine statistically different expression patterns between groups with p<0.05 considered statistically significant.
(TIF)

Figure S3 Scatterplot depicting the mRNA levels of the genes that were over-expressed in urinary bladder cancer (BC) versus normal tissue. Groups pairs were statistically compared using the Mann-Whitney U test. Bars depict the median values. **B.** Scatterplot depicting the mRNA levels of the genes that were equally expressed among urinary bladder cancer and normal tissue. Groups pairs were statistically compared using the Mann-Whitney U test. Bars depict the median values. **C.** Scatterplot depicting the mRNA levels of the genes that were under-expressed in urinary bladder cancer versus normal tissue. Groups pairs were statistically compared using the Mann-Whitney U test. Bars depict the median values.
(TIF)

Figure S4 Using the BioNetwork Tools from www.pubgene.org, relationships between the GOIs were investigated. HRAS confirmed its role as a key molecule, since it presented interaction with all the GOIs (19 interactions). OPN, TP53, BRAF and EGFR exhibited the second highest number of significant interactions (18 of the GOIs), whereas VEGFA, RAF1, CDKN2A. ki67, ERBB2 and FGF2 were associated with 17 of the GOIs. NRAS was associated with 16 GOIs, KRAS, MMP2 and MMP9 with 15 GOIs, TGFβ1 with 14 GOIs, ARAF with 13 GOIs, EGF with 6 GOIs and RKIP/PEBP1 with 5 of the GOIs.
(TIF)

Figure S5 HCL (**A**), PCA for experiments (**B**) and genes (**C**) as well as CA analyses (**D**) for the microarray dataset selected to be the same with the genes tested with qPCR. Colours of clusters in HCL correspond to the gene colours in all other analyses.
(TIF)

Table S1 Information of the primer sequences used for qPCR and the corresponding PCR product sizes.
(DOC)

Table S2 Gene expression analysis in urinary bladder cancer was performed on data extracted from the following publicly available datasets: GSE89, GSE3167, GSE7476 and GSE12630. T, tumor; M, metastasis, N, normal tissue; sTCC with CIS, superficial transitional cell carcinoma (sTCC) with surrounding carcinoma in situ (CIS); sTCC without CIS, superficial transitional cell carcinoma (sTCC) without surrounding carcinoma in situ (CIS); mTCC, muscle-invasive carcinomas (mTCC). Results were expressed as mean levels of the log_2_ intensity and statistically compared by the Mann-Whitney U test (p-values are reported for each comparison between expression levels in the tumor and normal cases).
(DOC)

Table S3 Fold differential change of each gene in BC vs. the control tissues. Red color indicates >2-fold expression; black color indicates equal expression with a threshold of 2; blue indicates <2-fold expression. T-test was performed between the expression levels of BC and control samples. The fold up- or down-regulation in BC vs. Control, was also explored using the mathematical formula: IF(Fold Difference>1; Fold Difference;-1/Fold Difference). If the fold change is positive or negative, it means up-or down-regulation, respectively.
(DOC)

Table S4 Spearman's bivariate correlation-coefficient test in order to detect correlations among the expression levels of all the genes studied in transitional cell carcinomas of the urinary bladder (**A**) and control tissue (**B**).
(DOC)

Table S5 Enriched KEGG pathways. This table lists the enriched KEGG pathways, number of Entrez IDs in the data set for the pathway, the corresponding Entrez IDs, and the statistics for the enrichment of the pathway. The statistics column lists the number of reference genes in the category (C), number of genes in the gene set and also in the category (O), expected number in the category (E), Ratio of enrichment (R), p value from hypergeometric test (rawP), and p value adjusted by the multiple test adjustment (adjP). The most significant pathway revealed, was the Bladder Cancer pathway. Interestingly, the most significant pathway revealed was Bladder Cancer pathway.
(DOC)
