# Supplementary figures and images for: Spotlight on Differentially Expressed Genes in Urinary Bladder Cancer
Source: PLoS One. 2011 Apr 5;6(4):e18255. doi: 10.1371/journal.pone.0018255 (PMC3071699; doi:10.1371/journal.pone.0018255)

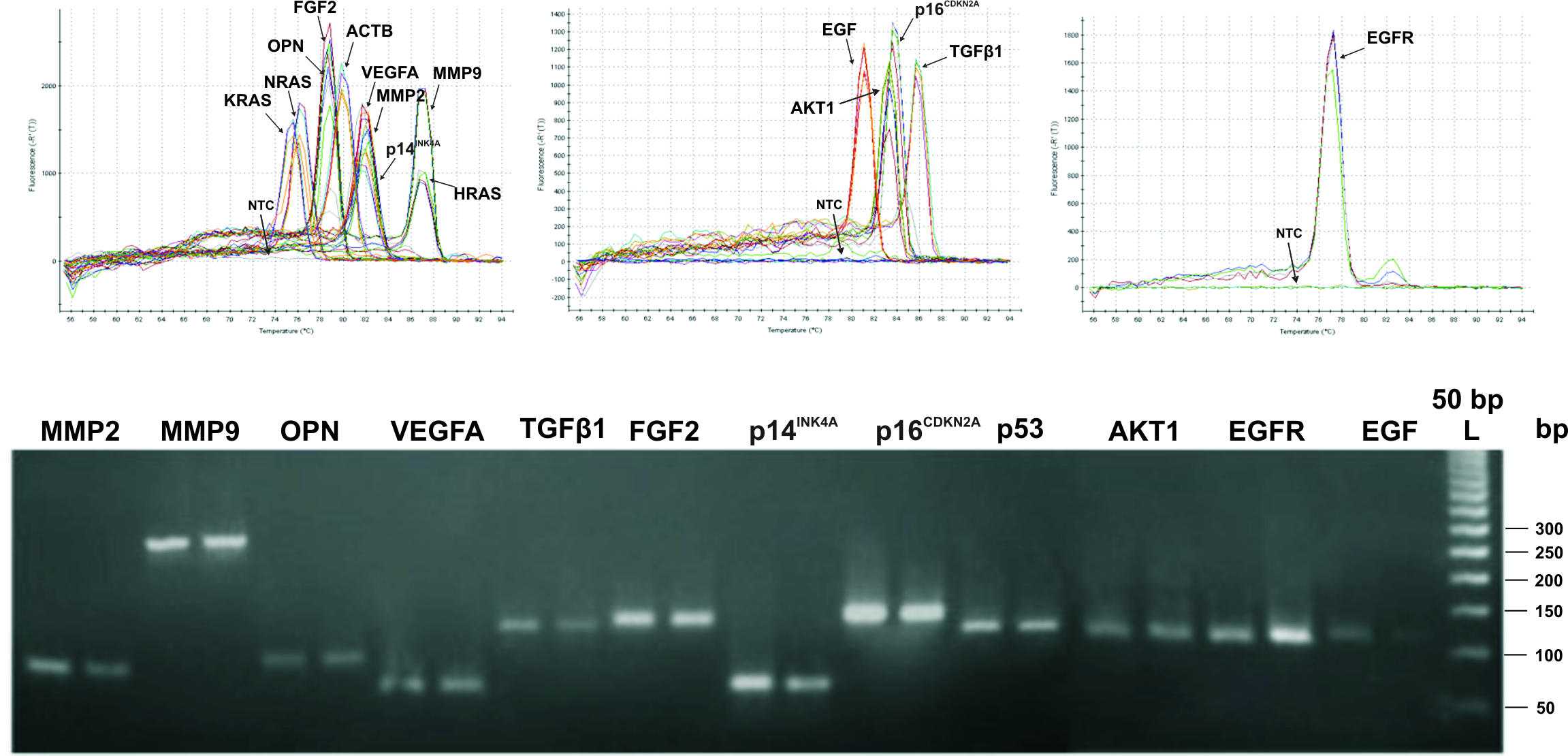

Supplement: Figure S1 — Upper panel: Melting curves used for product specificity of the genes MMP2, MMP9, OPN, VEGFA, TGFβ1, FGF2, p14ARF, p16INK4A, p53, AKT1, EGFR and EGF. NTC, non-template control. Lower panel: Representative examples of PCR products after analysis by electrophoresis on 2% agarose gel. (TIF) [file pone.0018255.s001.tif]

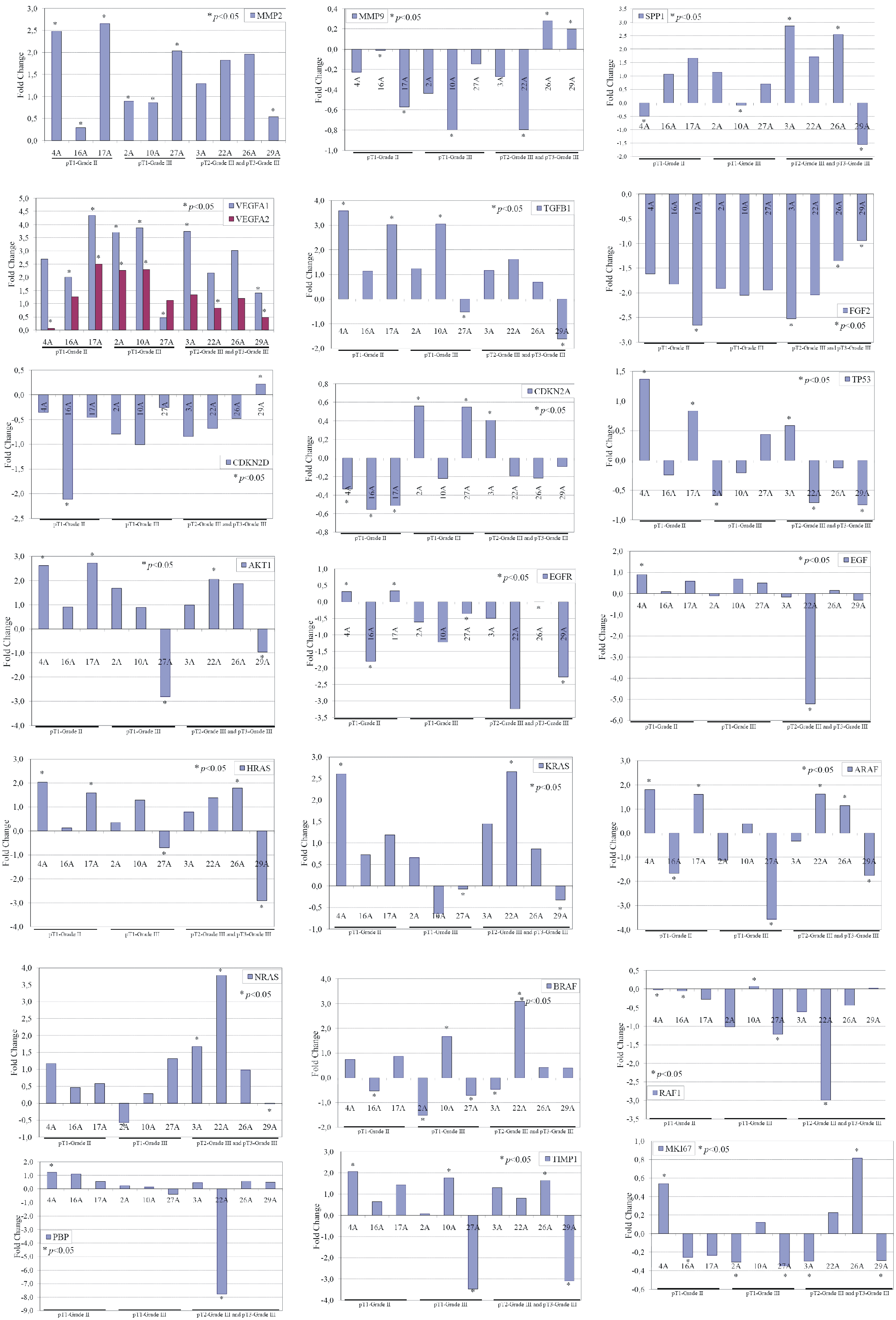

Supplement: Figure S2 — Differential fold expression of the genes of interest in 10 BC samples vs. 5 controls, as detected by microarray analysis. The Mann-Whitney test was performed to examine statistically different expression patterns between groups with p<0.05 considered statistically significant. (TIF) [file pone.0018255.s002.tif]

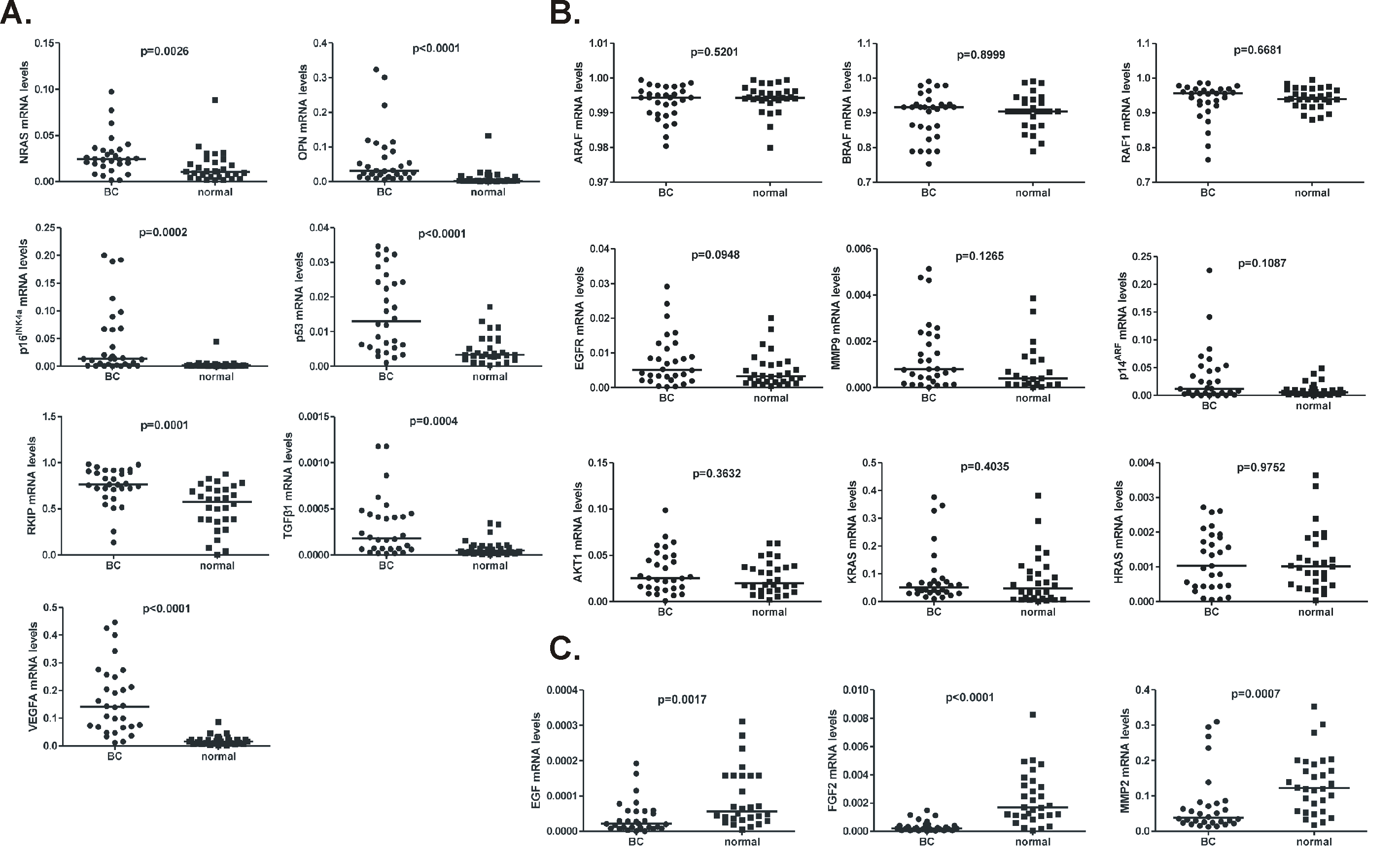

Supplement: Figure S3 — Scatterplot depicting the mRNA levels of the genes that were over-expressed in urinary bladder cancer (BC) versus normal tissue. Groups pairs were statistically compared using the Mann-Whitney U test. Bars depict the median values. B. Scatterplot depicting the mRNA levels of the genes that were equally expressed among urinary bladder cancer and normal tissue. Groups pairs were statistically compared using the Mann-Whitney U test. Bars depict the median values. C. Scatterplot depicting the mRNA levels of the genes that were under-expressed in urinary bladder cancer versus normal tissue. Groups pairs were statistically compared using the Mann-Whitney U test. Bars depict the median values. (TIF) [file pone.0018255.s003.tif]

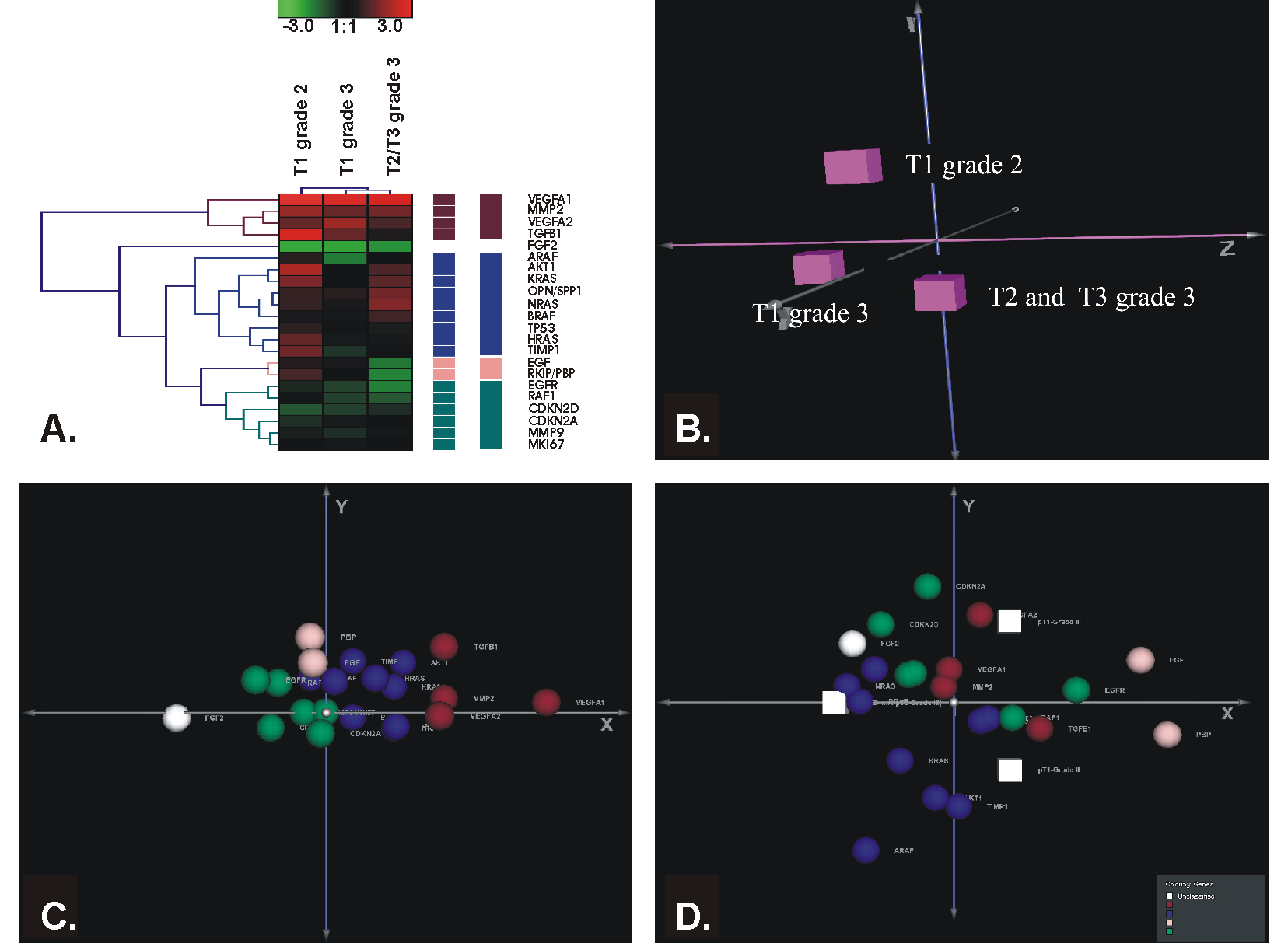

Supplement: Figure S5 — HCL (A), PCA for experiments (B) and genes (C) as well as CA analyses (D) for the microarray dataset selected to be the same with the genes tested with qPCR. Colours of clusters in HCL correspond to the gene colours in all other analyses. (TIF) [file pone.0018255.s005.tif]
