# Supplementary material for: Spotlight on Differentially Expressed Genes in Urinary Bladder Cancer
Source: PLoS One. 2011 Apr 5;6(4):e18255. doi: 10.1371/journal.pone.0018255 (PMC3071699; doi:10.1371/journal.pone.0018255)
Supplement: Table S1 — Information of the primer sequences used for qPCR and the corresponding PCR product sizes. (DOC) [file pone.0018255.s006.doc]

**Table S1.** Information of the primer sequences used for qPCR and the corresponding PCR product sizes.

| Gene name | GenBank AN | Primer Sequence | Annealing Temperature (ºC) | PCR product length (bp) |
| --- | --- | --- | --- | --- |
| KRAS | [NM_004985.3](http://www.ncbi.nlm.nih.gov/nuccore/NM_004985.3) | 5’-GGGGAGGGCTTTCTTTGTGTA-3’ (F) | 60 | 174 |
|  |  | 5’-GTCCTGAGCCTGTTTTGTGTC-3’ (R) |  |  |
| HRAS | [NM_001130442.1](http://www.ncbi.nlm.nih.gov/nuccore/NM_001130442.1) | 5’-GGGGCAGTCGCGCCTGTGAA-3’ (F) | 60 | 110 |
|  |  | 5’-CCGGCGCCCACCACCACCAG-3’ (R) |  |  |
| NRAS | [NM_002524.3](http://www.ncbi.nlm.nih.gov/nuccore/NM_002524.3) | 5’-CTTCCTCTGTGTATTTGCCATCA-3’ (F) | 60 | 107 |
|  |  | 5’-GCACCATAGGTACATCATCCGA-3’ (R) |  |  |
| IGF1 | [NM_000618.3](http://www.ncbi.nlm.nih.gov/nuccore/NM_000618.3) | 5’-CCTCCTCGCATCTCTTCTACCTGC-3’ (F) | 60 | 166 |
|  |  | 5’-TGCTGGAGCCATACCCTGTG-3’ (R) |  |  |
| TGFβ1 | [NM_000660.4](http://www.ncbi.nlm.nih.gov/nuccore/NM_000660.4) | 5’-AAGGACCTCGGCTGGAAGTGC-3’ (F) | 60 | 137 |
|  |  | 5’-CCGGGTTATGCTGGTTGTA-3’ (R) |  |  |
| VEGFA | [NM_001025366.2](http://www.ncbi.nlm.nih.gov/nuccore/NM_001025366.2) | 5’- ATGACGAGGGCCTGGAGTGTG-3’ (F) | 60 | 91 |
|  |  | 5’-CCTATGTGCTGGCCTTGGTGAG-3’ (R) |  |  |
| EGF | [NM_001178130.1](http://www.ncbi.nlm.nih.gov/nuccore/NM_001178130.1) | 5’-CTTGTCATGCTGCTCCTCCTG-3’ (F) | 60 | 118 |
|  |  | 5’-TGCGACTCCTCACATCTCTGC-3’ (R) |  |  |
| FGF2 | [NM_002006.4](http://www.ncbi.nlm.nih.gov/nuccore/NM_002006.4) | 5’-CTGGCTATGAAGGAAGATGGA-3’ (F) | 60 | 109 |
|  |  | 5’-TGCCCAGTTCGTTTCAGTG-3’ (R) |  |  |
| GAPDH | [NM_002046.3](http://www.ncbi.nlm.nih.gov/nuccore/NM_002046.3) | 5’-GGAAGGTGAAGGTCGGAGTCA-3’ (F) | 60 | 101 |
|  |  | 5’-GTCATTGATGGCAACAATATCCACT-3’ (R) |  |  |
| RPL13A | [NM_012423.2](http://www.ncbi.nlm.nih.gov/nuccore/NM_012423.2) | 5’-CCTGGAGGAGAAGAGGAAAGAGA-3’ (F) | 60 | 127 |
|  |  | 5’-TTGAGGACCTCTGTGTATTTGTCAA-3’ (R) |  |  |
| MMP2 | [NM_001127891.1](http://www.ncbi.nlm.nih.gov/nuccore/NM_001127891.1) | 5’-TGATCTTGACCAGAATACCATCGA-3’ (F) | 60 | 90 |
|  |  | 5’-GGCTTGCGAGGGAAGAAGT-3’ (R) |  |  |
| MMP9 | [NM_004994.2](http://www.ncbi.nlm.nih.gov/nuccore/NM_004994.2) | 5’-GTGCTGGGCTGCTGCTTTGCTG-3’ (F) | 60 | 303 |
|  |  | 5’-GTCGCCCTCAAAGGTTTGGAAT-3’ (R) |  |  |
| OPN (SPP1) | [NM_000582.2](http://www.ncbi.nlm.nih.gov/nuccore/NM_000582.2) | 5’-GCCGAGGTGATAGTGTGGTT-3’ (F) | 60 | 101 |
|  |  | 5’-TGAGGTGATGTCCTCGTCTG-3’ (R) |  |  |
| p14^ARF^ | [NM_000077.4](http://www.ncbi.nlm.nih.gov/nuccore/NM_000077.4) | 5’- CCCTCGTGCTGATGCTACTG-3’ (F) | 60 | 72 |
|  |  | 5’- CATCATGACCTGGTCTTCTAGGAA-3’ (R) |  |  |
| p16^INK4^ | [NM_058195.3](http://www.ncbi.nlm.nih.gov/nuccore/NM_058195.3) | 5’-GGGGGCACCAGAGGCAGT-3’ (F) | 60 | 159 |
|  |  | 5’-GGTTGTGGCGGGGGCAGTT-3’ (R) |  |  |
| p53 | [NM_000546.4](http://www.ncbi.nlm.nih.gov/nuccore/NM_000546.4) | 5’-GTGAGCGCTTCGAGATGTTC-3’ (F) | 60 | 137 |
|  |  | 5’-ATGGCGGGAGGTAGACTGAC-3’ (R) |  |  |
| AKT1 | [NM_001014431.1](http://www.ncbi.nlm.nih.gov/nuccore/NM_001014431.1) | 5’-GCACAAACGAGGGGAGTACAT-3’ (F) | 60 | 113 |
|  |  | 5’-CCTCACGTTGGTCCACATC-3’ (R) |  |  |
| ARAF | [NM_001654.3](http://www.ncbi.nlm.nih.gov/nuccore/NM_001654.3) | 5’-CGTCAAAGTATACCTGCCCAACA-3’ (F) | 56 | 107 |
|  |  | 5’-GATTTAGACCCCGCACCTTCA-3’ (R) |  |  |
| BRAF | [NM_004333.4](http://www.ncbi.nlm.nih.gov/nuccore/NM_004333.4) | 5’-AGAAAGCACTGATGATGAGAGG-3’ (F) | 53 | 101 |
|  |  | 5’-GGAAATATCAGTGTCCCAACCA-3’ (R) |  |  |
| RAF1 | [NM_002880.3](http://www.ncbi.nlm.nih.gov/nuccore/NM_002880.3) | 5’-ACTGCCTTATGAAAGCACTCAAG-3’ (F) | 57 | 120 |
|  |  | 5’-ACGCAGCATCAGTATTCCAAT-3’ (R) |  |  |
| RKIP | [NM_002567.2](http://www.ncbi.nlm.nih.gov/nuccore/NM_002567.2) | 5’-AGACCCACCAGCATTTCGTG-3’ (F) | 55 | 150 |
|  |  | 5’-GCTGATGTCATTGCCCTTCA-3’ (R) |  |  |
| EGFR | [NM_005228.3](http://www.ncbi.nlm.nih.gov/nuccore/NM_005228.3) | 5’-GCGTTCGGCACGGTGTATAA-3’ (F) | 60 | 102 |
|  |  | 5’-GGCTTTCGGAGATGTTGCTTC-3’ (R) |  |  |
