# Supplementary material for: Spotlight on Differentially Expressed Genes in Urinary Bladder Cancer
Source: PLoS One. 2011 Apr 5;6(4):e18255. doi: 10.1371/journal.pone.0018255 (PMC3071699; doi:10.1371/journal.pone.0018255)
Supplement: Table S2 — Gene expression analysis in urinary bladder cancer was performed on data extracted from the following publicly available datasets: GSE89, GSE3167, GSE7476 and GSE12630. T, tumor; M, metastasis, N, normal tissue; sTCC with CIS, superficial transitional cell carcinoma (sTCC) with surrounding carcinoma in situ (CIS); sTCC without CIS, superficial transitional cell carcinoma (sTCC) without surrounding carcinoma in situ (CIS); mTCC, muscle-invasive carcinomas (mTCC). Results were expressed as mean levels of the log2 intensity and statistically compared by the Mann-Whitney U test (p-values are reported for each comparison between expression levels in the tumor and normal cases). (DOC) [file pone.0018255.s007.doc]

| **Author, year,** | **Tissue type** | **Mean Log_2_ Transformed Ratios (Samples vs. Controls)** | | | | | | | | | | | | | | | | | | | | | | |
| --- | --- | --- | --- | --- | --- | --- | --- | --- | --- | --- | --- | --- | --- | --- | --- | --- | --- | --- | --- | --- | --- | --- | --- | --- |
| **GEO Dataset Ref. series** | **no. of samples** | **MMP2** | **MMP9** | **OPN/SPP1** | **VEGFA** | **TIMP1** | **TIMP2** | **TGFβ1** | **FGF2** | **p14^ARF^** | **p16^INK4A^** | **p53** | **AKT1** | **EGFR** | **EGF** | **Ki-67** | **KRAS** | **HRAS** | **NRAS** | **CyclinD1** | **ARAF** | **BRAF** | **RAF1** | **RKIP** |
| **Dyrskjøt et al., 2003, GSE89** | **T1, grade 3 (n=11)** | **-0.81** | **-3.07** | **-1.80** | **-0.48** | **-6.17** | **-1.24** | **1.42** | **-2.43** | **-0.27** | **0.55** | **1.09** | **0.61** | **-5.02** | **-0.89** | **-2.03** | **-2.37** | **1.31** | **-1.88** | **3.30** | **-1.06** | **-0.08** | **0.41** | **1.03** |
|  | **T2+, grade 3 (n=9)** | **-0.15** | **1.63** | **1.08** | **-0.50** | **-6.93** | **0.69** | **1.66** | **-3.10** | **0.23** | **2.13** | **0.12** | **0.76** | **-3.18** | **-1.79** | **-0.84** | **-2.75** | **1.40** | **-2.97** | **1.46** | **-0.92** | **-0.41** | **0.44** | **0.32** |
|  | **Ta, grade 2 (n=6)** | **-0.83** | **-2.86** | **-1.99** | **-0.43** | **-5.73** | **-1.17** | **1.59** | **-3.18** | **0.02** | **1.00** | **1.80** | **0.98** | **-3.14** | **-2.43** | **-2.54** | **-2.42** | **1.39** | **-1.92** | **3.50** | **-0.89** | **-0.04** | **0.03** | **1.44** |
|  | **Ta, grade 3 (n=14)** | **-0.84** | **-2.45** | **-2.15** | **-0.77** | **-6.10** | **-0.68** | **0.67** | **-4.06** | **0.37** | **0.22** | **1.77** | **0.88** | **-3.16** | **-2.87** | **-2.46** | **-2.04** | **1.46** | **-0.57** | **2.46** | **-0.88** | **0.08** | **0.92** | **0.87** |
|  | **Normal (n=9)** |  |  |  |  |  |  |  |  |  |  |  |  |  |  |  |  |  |  |  |  |  |  |  |
| **Mengual et al., 2009, GSE7476** | **Ta, low grade (n=3)** | **-0.31** | **-0.06** | **0.12** | **0.04** | **-0.08** | **-0.40** | **0.04** | **-0.44** | **0.11** | **0.05** | **0.20** | **0.07** | **0.08** | **0.08** | **0.14** | **0.08** | **0.13** | **0.22** | **0.21** | **0.02** | **0.12** | **-0.64** | **0.03** |
|  | **T1, high grade (n=3)** | **-0.28** | **0.22** | **0.10** | **0.13** | **-0.08** | **-0.17** | **0.00** | **-0.44** | **0.04** | **0.05** | **0.06** | **0.01** | **0.14** | **0.14** | **0.40** | **0.15** | **0.04** | **0.19** | **0.12** | **0.01** | **0.21** | **-0.49** | **0.11** |
|  | **T2, T3 or T4 (high grade) (n=3)** | **-0.21** | **0.17** | **0.18** | **0.09** | **-0.05** | **0.03** | **0.02** | **-0.38** | **0.02** | **0.07** | **0.17** | **0.03** | **0.19** | **0.19** | **0.52** | **0.30** | **0.02** | **0.21** | **0.29** | **0.03** | **0.10** | **-0.48** | **0.06** |
|  | **Normal (n=3)** |  |  |  |  |  |  |  |  |  |  |  |  |  |  |  |  |  |  |  |  |  |  |  |
| **Dyrskjøt et al., 2004, GSE3167** | **sTCC with CIS (n=13)** | **-0.34** | **-0.04** | **-0.36** | **2.43** | **0.24** | **-0.55** | **-0.71** | **-1.33** | **-0.55** | **-0.81** | **0.44** | **0.65** | **-0.94** | **-0.97** | **-0.34** | **1.84** | **0.78** | **0.17** | **1.63** | **-0.01** | **-0.36** | **1.29** | **1.01** |
|  | **sTCC without CIS (n=15)** | **-0.76** | **-0.14** | **-0.79** | **1.90** | **0.03** | **-0.49** | **-0.13** | **-0.88** | **-0.13** | **-0.24** | **0.71** | **0.75** | **-0.33** | **-0.71** | **-0.28** | **0.45** | **1.21** | **0.03** | **1.99** | **0.28** | **-0.26** | **0.94** | **1.23** |
|  | **mTCC (n=13)** | **0.60** | **2.27** | **2.28** | **2.11** | **1.20** | **0.40** | **-0.31** | **-1.12** | **-0.36** | **-0.41** | **0.46** | **0.67** | **-0.38** | **-0.79** | **-0.12** | **1.03** | **0.87** | **0.30** | **1.52** |  | **-0.57** | **0.78** | **0.56** |
|  | **cystectomy specimens (n=10)** | **0.35** | **0.52** | **-0.99** | **2.21** | **0.57** | **-0.17** | **-0.45** | **-0.04** | **-0.36** | **-0.59** | **-0.45** | **-0.08** | **-0.49** | **-0.26** | **-0.19** | **0.72** | **-0.26** | **-0.25** | **0.51** | **-0.39** | **0.05** | **0.37** | **-0.21** |
|  | **Normal (n=9)** |  |  |  |  |  |  |  |  |  |  |  |  |  |  |  |  |  |  |  |  |  |  |  |
| **Monzon et al., 2009, GSE12630** | **TCC of the urinary bladder (n=13)** | **1.26** | **0.71** | **1.24** | **1.21** | **1.73** | **0.49** | **-0.55** | **-0.25** | **-0.97** | **-0.92** | **0.39** | **0.56** | **-0.12** | **-0.95** | **0.39** | **1.14** | **0.57** | **0.62** | **0.60** | **0.60** | **0.24** | **0.90** | **1.01** |
|  | **Metastatic high grade (n=8)** | **0.87** | **1.06** | **0.81** | **1.07** | **1.58** | **0.17** | **-0.56** | **-0.69** | **-1.12** | **-0.65** | **0.47** | **0.78** | **-0.27** | **-0.84** | **0.36** | **1.00** | **0.76** | **0.35** | **0.48** | **0.67** | **0.30** | **0.92** | **1.19** |
| **Zaravinos et al., 2011, GSE27448** | **pT1-Grade II (n=3)** | **1.81** | **-0.27** | **0.75** | **3.01** | **1.38** | **N/A** | **2.58** | **-2.03** | **-0.98** | **-0.47** | **0.65** | **2.08** | **-0.39** | **0.53** | **0.02** | **1.51** | **1.25** | **0.74** | **N/A** | **0.58** | **0.36** | **-0.12** | **0.95** |
|  | **pT1-Grade III (n=3)** | **1.26** | **-0.46** | **0.58** | **2.68** | **-0.55** | **N/A** | **1.26** | **-1.97** | **-0.69** | **0.30** | **-0.12** | **-0.08** | **-0.73** | **0.36** | **-0.18** | **-0.02** | **0.32** | **0.34** | **N/A** | **-1.44** | **-0.19** | **-0.72** | **-0.01** |
|  | **pT2- and pT3-Grade III (n=4)** | **1.40** | **-0.15** | **1.39** | **2.58** | **0.17** | **N/A** | **0.47** | **-1.72** | **-0.45** | **-0.02** | **-0.25** | **0.99** | **-1.50** | **-1.38** | **0.11** | **1.16** | **0.26** | **1.60** | **N/A** | **0.17** | **0.86** | **-1.00** | **-1.56** |
|  | **Normal (n=5)** |  |  |  |  |  |  |  |  |  |  |  |  |  |  |  |  |  |  |  |  |  |  |  |

**Table S2.** Gene expression analysis in urinary bladder cancer was performed on data extracted from the following publicly available datasets: GSE89, GSE3167, GSE7476 and GSE12630. T, tumor; M, metastasis, N, normal tissue; sTCC with CIS, superficial transitional cell carcinoma (sTCC) with surrounding carcinoma in situ (CIS); sTCC without CIS, superficial transitional cell carcinoma (sTCC) without surrounding carcinoma in situ (CIS); mTCC, muscle-invasive carcinomas (mTCC). Results were expressed as mean levels of the log_2_ intensity and statistically compared by the Mann-Whitney U test (p-values are reported for each comparison between expression levels in the tumor and normal cases).
