# Supplementary material for: Spotlight on Differentially Expressed Genes in Urinary Bladder Cancer
Source: PLoS One. 2011 Apr 5;6(4):e18255. doi: 10.1371/journal.pone.0018255 (PMC3071699; doi:10.1371/journal.pone.0018255)
Supplement: Table S3 — Fold differential change of each gene in BC vs. the control tissues. Red color indicates >2-fold expression; black color indicates equal expression with a threshold of 2; blue indicates <2-fold expression. T-test was performed between the expression levels of BC and control samples. The fold up- or down-regulation in BC vs. Control, was also explored using the mathematical formula: IF(Fold Difference>1; Fold Difference;-1/Fold Difference). If the fold change is positive or negative, it means up-or down-regulation, respectively. (DOC) [file pone.0018255.s008.doc]

|  | **2^-ΔCt** | | **Fold Difference** | **T-TEST** | **Fold Up- or Down-Regulation** |
| --- | --- | --- | --- | --- | --- |
|  | **BC** | **Control** | **BC/Control** | **p value** | **BC/Control** |
| **MMP2** | 3,4E-02 | 8,4E-02 | 0,41 | 0,0000937776 | -2,45 |
| **MMP9** | 3,7E-04 | 2,7E-04 | 1,37 | 0,0000006489 | 1,37 |
| **OPN** | 2,3E-02 | 1,8E-03 | 12,47 | 0,0000003867 | 12,47 |
| **VEGFA** | 1,1E-01 | 1,4E-02 | 8,17 | 0,0000031485 | 8,17 |
| **TGFβ1** | 1,6E-04 | 4,5E-05 | 3,63 | 0,0000219967 | 3,63 |
| **FGF2** | 1,9E-04 | 1,5E-03 | 0,13 | 0,0000034562 | -7,78 |
| **p14^ARF^** | 7,0E-03 | 5,5E-03 | 1,27 | 0,0127804838 | 1,27 |
| **p16^INK4A^** | 1,1E-02 | 1,4E-03 | 7,41 | 0,0000038025 | 7,41 |
| **p53** | 1,1E-02 | 3,4E-03 | 3,27 | 0,0000307193 | 3,27 |
| **AKT1** | 2,3E-02 | 2,0E-02 | 1,16 | 0,0544069998 | 1,16 |
| **EGFR** | 4,6E-03 | 3,3E-03 | 1,40 | 0,0038795741 | 1,40 |
| **EGF** | 1,9E-05 | 7,3E-05 | 0,25 | 0,0000171554 | -3,94 |
| **HRAS** | 8,4E-04 | 9,3E-04 | 0,91 | 0,8211990468 | -1,10 |
| **KRAS** | 5,7E-02 | 3,5E-02 | 1,60 | 0,0011357999 | 1,60 |
| **NRAS** | 2,4E-02 | 9,8E-03 | 2,39 | 0,0001029024 | 2,39 |
| **ARAF** | 9,9E-01 | 9,9E-01 | 1,00 | 1,0000000000 | -1,00 |
| **BRAF** | 8,9E-01 | 9,0E-01 | 0,99 | 0,0704839969 | -1,01 |
| **RAF1** | 9,3E-01 | 9,4E-01 | 0,99 | 0,1599022207 | -1,01 |
| **RKIP** | 7,0E-01 | 3,9E-01 | 1,82 | 0,0000000487 | 1,82 |

**Table S3.** Fold differential change of each gene in BC versus the control tissues. Red color indicates >2-fold expression; black color indicates equal expression with a threshold of 2; blue indicates <2-fold expression. T-test was performed between the expression levels of BC and control samples. The fold up- or down-regulation in BC vs. Control, was also explored using the mathematical formula: IF(Fold Difference>1; Fold Difference;-1/ Fold Difference). If the fold change is positive or negative, it means up-or down-regulation, respectively.
