# Supplementary material for: Spotlight on Differentially Expressed Genes in Urinary Bladder Cancer
Source: PLoS One. 2011 Apr 5;6(4):e18255. doi: 10.1371/journal.pone.0018255 (PMC3071699; doi:10.1371/journal.pone.0018255)
Supplement: Table S4 — Spearman's bivariate correlation-coefficient test in order to detect correlations among the expression levels of all the genes studied in transitional cell carcinomas of the urinary bladder (A) and control tissue (B). (DOC) [file pone.0018255.s009.doc]

**Table S4.** Spearman’s bivariate correlation-coefficient test in order to detect correlations among the expression levels of all the genes studied in transitional cell carcinomas of the urinary bladder (**A**) and control tissue (**B**).

| **(A)** |  | MMP2 | MMP9 | OPN | VEGFA | TGFB1 | FGF2 | p14^ARF^ | p16^INK4A^ | p53 | AKT1 | EGFR | EGF | HRAS | KRAS | NRAS | ARAF | BRAF | RAF1 | RKIP |
| --- | --- | --- | --- | --- | --- | --- | --- | --- | --- | --- | --- | --- | --- | --- | --- | --- | --- | --- | --- | --- |
| MMP2 | CC | 1.000 |  |  |  |  |  |  |  |  |  |  |  |  |  |  |  |  |  |  |
|  | p-value |  |  |  |  |  |  |  |  |  |  |  |  |  |  |  |  |  |  |  |
| MMP9 | CC | .149 | 1.000 |  |  |  |  |  |  |  |  |  |  |  |  |  |  |  |  |  |
|  | p-value | .432 |  |  |  |  |  |  |  |  |  |  |  |  |  |  |  |  |  |  |
| OPN | CC | -.313 | .186 | 1.000 |  |  |  |  |  |  |  |  |  |  |  |  |  |  |  |  |
|  | p-value | .092 | .326 |  |  |  |  |  |  |  |  |  |  |  |  |  |  |  |  |  |
| VEGFA | CC | -.130 | -.502(**) | .342 | 1.000 |  |  |  |  |  |  |  |  |  |  |  |  |  |  |  |
|  | p-value | .495 | **.005** | .064 |  |  |  |  |  |  |  |  |  |  |  |  |  |  |  |  |
| TGFB1 | CC | -.259 | -.205 | .220 | .284 | 1.000 |  |  |  |  |  |  |  |  |  |  |  |  |  |  |
|  | p-value | .167 | .277 | .242 | .128 |  |  |  |  |  |  |  |  |  |  |  |  |  |  |  |
| FGF2_ | CC | .089 | .438(*) | .015 | -.426(*) | .154 | 1.000 |  |  |  |  |  |  |  |  |  |  |  |  |  |
|  | p-value | .640 | .016 | .936 | **.019** | .416 |  |  |  |  |  |  |  |  |  |  |  |  |  |  |
| p14^ARF^ | CC | -.021 | .008 | -.044 | .079 | -.256 | -.095 | 1.000 |  |  |  |  |  |  |  |  |  |  |  |  |
|  | p-value | .914 | .966 | .816 | .680 | .172 | .619 |  |  |  |  |  |  |  |  |  |  |  |  |  |
| p16^INK4A^ | CC | -.173 | .180 | .006 | -.110 | -.169 | .175 | .685(**) | 1.000 |  |  |  |  |  |  |  |  |  |  |  |
|  | p-value | .360 | .341 | .975 | .562 | .373 | .355 | **.000** |  |  |  |  |  |  |  |  |  |  |  |  |
| p53 | CC | .067 | -.177 | .220 | .259 | .390(*) | .207 | .148 | .186 | 1.000 |  |  |  |  |  |  |  |  |  |  |
|  | p-value | .725 | .348 | .243 | .167 | **.033** | .272 | .437 | .326 |  |  |  |  |  |  |  |  |  |  |  |
| AKT1 | CC | .166 | .231 | .081 | -.105 | .021 | .329 | .089 | .130 | .620(**) | 1.000 |  |  |  |  |  |  |  |  |  |
|  | p-value | .380 | .220 | .670 | .582 | .912 | .076 | .638 | .492 | **.000** |  |  |  |  |  |  |  |  |  |  |
| EGFR | CC | .131 | .329 | .236 | -.176 | .364(*) | .440(*) | .095 | .171 | .551(**) | .625(**) | 1.000 |  |  |  |  |  |  |  |  |
|  | p-value | .490 | .076 | .208 | .354 | **.048** | **.015** | .618 | .367 | **.002** | .000 |  |  |  |  |  |  |  |  |  |
| EGF | CC | .012 | .230 | .045 | -.110 | -.134 | .023 | .251 | .433(*) | .001 | .057 | .158 | 1.000 |  |  |  |  |  |  |  |
|  | p-value | .950 | .220 | .815 | .561 | .480 | .903 | .181 | **.017** | .995 | .765 | .403 |  |  |  |  |  |  |  |  |
| HRAS | CC | -.169 | -.311 | .233 | .324 | .020 | -.497(**) | .353 | .206 | .112 | -.017 | .089 | .017 | 1.000 |  |  |  |  |  |  |
|  | p-value | .371 | .094 | .215 | .081 | .914 | **.005** | .056 | .276 | .555 | .927 | .639 | .927 |  |  |  |  |  |  |  |
| KRAS | CC | .128 | -.225 | .084 | .360 | -.114 | -.399(*) | .435(*) | .013 | .190 | .116 | -.125 | .093 | .356 | 1.000 |  |  |  |  |  |
|  | p-value | .501 | .233 | .660 | .051 | .550 | **.029** | .016 | .944 | .315 | .543 | .510 | .624 | .053 |  |  |  |  |  |  |
| NRAS | CC | -.329 | -.082 | .405(*) | .245 | -.019 | -.095 | .470(**) | .539(**) | .327 | .099 | .163 | .404(*) | .480(**) | .232 | 1.000 |  |  |  |  |
|  | p-value | .076 | .668 | **.027** | .191 | .921 | .618 | **.009** | **.002** | .077 | .602 | .389 | **.027** | **.007** | .218 |  |  |  |  |  |
| ARAF | CC | .335 | .044 | -.153 | -.217 | -.008 | .319 | -.371(*) | -.315 | .110 | .183 | .166 | -.307 | -.397(*) | -.310 | -.524(**) | 1.000 |  |  |  |
|  | p-value | .070 | .818 | .421 | .250 | .966 | .086 | **.044** | .090 | .563 | .332 | .382 | .099 | **.030** | .095 | **.003** |  |  |  |  |
| BRAF | CC | .393(*) | -.139 | -.160 | -.255 | .076 | .235 | -.357 | -.235 | -.024 | -.002 | .095 | -.222 | -.189 | -.105 | -.505(**) | .554(**) | 1.000 |  |  |
|  | p-value | **.032** | .465 | .397 | .174 | .692 | .211 | .053 | .211 | .901 | .991 | .619 | .238 | .318 | .582 | .004 | .001 |  |  |  |
| RAF1 | CC | .378(*) | -.020 | -.171 | -.105 | .307 | .273 | -.278 | -.335 | .375(*) | .102 | .271 | -.290 | -.197 | .011 | -.325 | .566(**) | .377(*) | 1.000 |  |
|  | p-value | **.039** | .914 | .365 | .580 | .099 | .144 | .136 | .070 | **.041** | .591 | .147 | .120 | .297 | .954 | .079 | **.001** | **.040** |  |  |
| RKIP | CC | -.445(*) | -.118 | .028 | .320 | .392(*) | .033 | -.081 | .091 | -.039 | -.418(*) | -.053 | .018 | -.079 | -.386(*) | .031 | .025 | -.289 | .029 | 1.000 |
|  | p-value | **.014** | .536 | .885 | .084 | **.032** | .862 | .670 | .631 | .838 | **.022** | .780 | .926 | .679 | **.035** | .873 | .897 | .121 | .880 |  |
| **(B)** |  |  |  |  |  |  |  |  |  |  |  |  |  |  |  |  |  |  |  |  |
| MMP2 | CC | 1.000 |  |  |  |  |  |  |  |  |  |  |  |  |  |  |  |  |  |  |
|  | p-value |  |  |  |  |  |  |  |  |  |  |  |  |  |  |  |  |  |  |  |
| MMP9 | CC | .267 | 1.000 |  |  |  |  |  |  |  |  |  |  |  |  |  |  |  |  |  |
|  | p-value | .207 |  |  |  |  |  |  |  |  |  |  |  |  |  |  |  |  |  |  |
| OPN | CC | -.014 | .596(**) | 1.000 |  |  |  |  |  |  |  |  |  |  |  |  |  |  |  |  |
|  | p-value | .941 | **.002** |  |  |  |  |  |  |  |  |  |  |  |  |  |  |  |  |  |
| VEGFA | CC | -.049 | .096 | .315 | 1.000 |  |  |  |  |  |  |  |  |  |  |  |  |  |  |  |
|  | p-value | .795 | .655 | .096 |  |  |  |  |  |  |  |  |  |  |  |  |  |  |  |  |
| TGFB1 | CC | -.235 | .265 | .104 | .391(*) | 1.000 |  |  |  |  |  |  |  |  |  |  |  |  |  |  |
|  | p-value | .212 | .210 | .592 | .033 |  |  |  |  |  |  |  |  |  |  |  |  |  |  |  |
| FGF2_ | CC | -.193 | -.028 | -.127 | .061 | .641(**) | 1.000 |  |  |  |  |  |  |  |  |  |  |  |  |  |
|  | p-value | .308 | .896 | .510 | .749 | **.000** |  |  |  |  |  |  |  |  |  |  |  |  |  |  |
| p14^ARF^ | CC | .707(**) | .100 | .033 | -.016 | -.537(**) | -.423(*) | 1.000 |  |  |  |  |  |  |  |  |  |  |  |  |
|  | p-value | **.000** | .642 | .863 | .933 | **.002** | .020 |  |  |  |  |  |  |  |  |  |  |  |  |  |
| p16^INK4A^ | CC | -.136 | .161 | .488(**) | .690(**) | .533(**) | .242 | -.118 | 1.000 |  |  |  |  |  |  |  |  |  |  |  |
|  | p-value | .472 | .453 | **.007** | **.000** | **.002** | .198 | .536 |  |  |  |  |  |  |  |  |  |  |  |  |
| p53 | CC | .000 | .277 | .226 | .647(**) | .543(**) | .270 | .081 | .622(**) | 1.000 |  |  |  |  |  |  |  |  |  |  |
|  | p-value | .998 | .201 | .248 | **.000** | **.002** | .157 | .678 | **.000** |  |  |  |  |  |  |  |  |  |  |  |
| AKT1 | CC | .715(**) | .273 | .010 | -.047 | -.209 | -.071 | .641(**) | -.250 | .235 | 1.000 |  |  |  |  |  |  |  |  |  |
|  | p-value | **.000** | .197 | .961 | .807 | .267 | .710 | **.000** | .183 | .219 |  |  |  |  |  |  |  |  |  |  |
| EGFR | CC | .119 | .071 | -.129 | .228 | .380(*) | .535(**) | -.071 | .100 | .577(**) | .520(**) | 1.000 |  |  |  |  |  |  |  |  |
|  | p-value | .529 | .741 | .506 | .225 | **.038** | **.002** | .708 | .598 | **.001** | **.003** |  |  |  |  |  |  |  |  |  |
| EGF | CC | .681(**) | -.011 | -.008 | -.165 | -.466(**) | -.358 | .509(**) | -.349 | -.242 | .518(**) | .062 | 1.000 |  |  |  |  |  |  |  |
|  | p-value | **.000** | .958 | .969 | .383 | **.009** | .052 | **.004** | .059 | .206 | **.003** | .746 |  |  |  |  |  |  |  |  |
| HRAS | CC | .280 | .011 | .044 | .202 | .145 | -.045 | -.075 | .232 | .026 | .000 | .004 | .293 | 1.000 |  |  |  |  |  |  |
|  | p-value | .134 | .958 | .819 | .284 | .445 | .812 | .694 | .218 | .893 | .998 | .983 | .116 |  |  |  |  |  |  |  |
| KRAS | CC | .377(*) | -.057 | -.206 | .042 | -.406(*) | -.357 | .520(**) | -.126 | -.023 | .389(*) | .006 | .128 | .044 | 1.000 |  |  |  |  |  |
|  | p-value | **.040** | .790 | .284 | .824 | **.026** | .053 | **.003** | .506 | .906 | **.034** | .975 | .500 | .819 |  |  |  |  |  |  |
| NRAS | CC | -.516(**) | .351 | .376(*) | .504(**) | .538(**) | .250 | -.403(*) | .655(**) | .542(**) | -.406(*) | .052 | -.608(**) | .041 | -.055 | 1.000 |  |  |  |  |
|  | p-value | **.003** | .093 | .045 | .005 | .002 | .182 | **.027** | **.000** | **.002** | **.026** | .786 | **.000** | .829 | .773 |  |  |  |  |  |
| ARAF | CC | .554(**) | .157 | .000 | -.209 | -.446(*) | -.321 | .663(**) | -.270 | -.053 | .637(**) | .054 | .459(*) | -.321 | .044 | -.650(**) | 1.000 |  |  |  |
|  | p-value | **.001** | .464 | .999 | .267 | **.013** | .083 | **.000** | .149 | .784 | .**000** | .776 | **.011** | .083 | .817 | **.000** |  |  |  |  |
| BRAF | CC | .317 | .449(*) | .032 | -.353 | -.480(**) | -.419(*) | .423(*) | -.458(*) | -.273 | .379(*) | -.192 | .254 | -.389(*) | .028 | -.451(*) | .709(**) | 1.000 |  |  |
|  | p-value | .088 | **.028** | .869 | .056 | **.007** | **.021** | **.020** | **.011** | .151 | **.039** | .310 | .175 | **.033** | .883 | **.012** | **.000** |  |  |  |
| RAF1 | CC | .110 | .082 | .028 | -.294 | -.306 | -.117 | .272 | -.358 | -.248 | .236 | -.113 | .122 | -.410(*) | -.300 | -.516(**) | .698(**) | .627(**) | 1.000 |  |
|  | p-value | .562 | .702 | .884 | .115 | .100 | .539 | .145 | .052 | .195 | .210 | .550 | .521 | **.024** | .107 | **.004** | **.000** | **.000** |  |  |
| RKIP | CC | -.405(*) | .156 | .118 | .187 | .330 | .199 | -.436(*) | .255 | .148 | -.407(*) | -.128 | -.267 | .101 | -.428(*) | .480(**) | -.457(*) | -.188 | -.178 | 1.000 |
|  | p-value | **.026** | .468 | .543 | .321 | .075 | .292 | **.016** | .174 | .442 | **.025** | .499 | .153 | .594 | **.018** | **.007** | **.011** | .319 | .346 |  |
