# Supplementary material for: Spotlight on Differentially Expressed Genes in Urinary Bladder Cancer
Source: PLoS One. 2011 Apr 5;6(4):e18255. doi: 10.1371/journal.pone.0018255 (PMC3071699; doi:10.1371/journal.pone.0018255)
Supplement: Table S5 — Enriched KEGG pathways. This table lists the enriched KEGG pathways, number of Entrez IDs in the data set for the pathway, the corresponding Entrez IDs, and the statistics for the enrichment of the pathway. The statistics column lists the number of reference genes in the category (C), number of genes in the gene set and also in the category (O), expected number in the category (E), Ratio of enrichment (R), p value from hypergeometric test (rawP), and p value adjusted by the multiple test adjustment (adjP). The most significant pathway revealed, was the Bladder Cancer pathway. Interestingly, the most significant pathway revealed was Bladder Cancer pathway. (DOC) [file pone.0018255.s010.doc]

**Table S5**. Enriched KEGG pathways. This table lists the enriched KEGG pathways, number of Entrez IDs in the data set for the pathway, the corresponding Entrez IDs, and the statistics for the enrichment of the pathway. The statistics column lists the number of reference genes in the category (C), number of genes in the gene set and also in the category (O), expected number in the category (E), Ratio of enrichment (R), p value from hypergeometric test (rawP), and p value adjusted by the multiple test adjustment (adjP). The most significant pathway revealed, was the Bladder Cancer pathway. Interestingly, the most significant pathway revealed was Bladder Cancer pathway.

| **Enriched KEGG pathways** | | |
| --- | --- | --- |
| Bladder cancer | 4318 4893 673 369 5894 4313 1950 7157 3845 3265 1029 1956 | C=42;O=12;E=0.02;R=681.79;rawP=3.53e-33;adjP=1.59e-31 |
| Melanoma | 4893 673 369 207 2247 5894 1950 7157 3845 3265 1029 1956 | C=71;O=12;E=0.03;R=403.31;rawP=4.07e-30;adjP=9.16e-29 |
| Pathways in cancer | 4318 7040 4893 673 369 207 2247 5894 4313 1950 7157 3845 3265 1029 1956 | C=330;O=15;E=0.14;R=108.47;rawP=2.34e-29;adjP=3.51e-28 |
| Non-small cell lung cancer | 4893 673 369 207 5894 1950 7157 3845 3265 1029 1956 | C=54;O=11;E=0.02;R=486.09;rawP=1.73e-28;adjP=1.95e-27 |
| Glioma | 4893 673 369 207 5894 1950 7157 3845 3265 1029 1956 | C=65;O=11;E=0.03;R=403.83;rawP=1.61e-27;adjP=1.45e-26 |
| Endometrial cancer | 4893 673 369 207 5894 1950 7157 3845 3265 1956 | C=52;O=10;E=0.02;R=458.90;rawP=1.44e-25;adjP=1.08e-24 |
| Pancreatic cancer | 7040 673 369 207 5894 1950 7157 3845 1029 1956 | C=72;O=10;E=0.03;R=331.43;rawP=4.85e-24;adjP=3.12e-23 |
| Chronic myeloid leukemia | 7040 4893 673 369 207 5894 7157 3845 3265 1029 | C=75;O=10;E=0.03;R=318.17;rawP=7.49e-24;adjP=4.21e-23 |
| Prostate cancer | 4893 673 369 207 5894 1950 7157 3845 3265 1956 | C=89;O=10;E=0.04;R=268.12;rawP=4.58e-23;adjP=2.29e-22 |
| MAPK signaling pathway | 7040 4893 673 207 2247 5894 1950 7157 3845 3265 1956 | C=269;O=11;E=0.11;R=97.58;rawP=1.89e-20;adjP=8.50e-20 |
| ErbB signaling pathway | 4893 673 369 207 5894 1950 3845 3265 1956 | C=87;O=9;E=0.04;R=246.85;rawP=2.09e-20;adjP=8.55e-20 |
| Renal cell carcinoma | 7040 4893 673 369 207 5894 3845 3265 | C=70;O=8;E=0.03;R=272.72;rawP=1.59e-18;adjP=5.96e-18 |
| Colorectal cancer | 7040 673 369 207 5894 7157 3845 1956 | C=84;O=8;E=0.04;R=227.26;rawP=7.32e-18;adjP=2.53e-17 |
| Regulation of actin cytoskeleton | 4893 673 369 2247 5894 1950 3845 3265 1956 | C=216;O=9;E=0.09;R=99.43;rawP=9.47e-17;adjP=3.04e-16 |
| Acute myeloid leukemia | 3845 4893 673 369 207 3265 5894 | C=60;O=7;E=0.03;R=278.40;rawP=2.46e-16;adjP=7.38e-16 |
| Neurotrophin signaling pathway | 3845 4893 673 207 3265 5894 7157 | C=126;O=7;E=0.05;R=132.57;rawP=5.30e-14;adjP=1.49e-13 |
| Insulin signaling pathway | 3845 4893 673 369 207 3265 5894 | C=137;O=7;E=0.06;R=121.93;rawP=9.63e-14;adjP=2.55e-13 |
| Long-term potentiation | 3845 4893 673 369 3265 5894 | C=70;O=6;E=0.03;R=204.54;rawP=2.90e-13;adjP=6.87e-13 |
| Long-term depression | 3845 4893 673 369 3265 5894 | C=70;O=6;E=0.03;R=204.54;rawP=2.90e-13;adjP=6.87e-13 |
| Thyroid cancer | 3845 4893 673 3265 7157 | C=29;O=5;E=0.01;R=411.42;rawP=8.60e-13;adjP=1.94e-12 |
| Gap junction | 3845 4893 3265 5894 1956 1950 | C=90;O=6;E=0.04;R=159.08;rawP=1.37e-12;adjP=2.94e-12 |
| Focal adhesion | 6696 673 207 3265 5894 1956 1950 | C=201;O=7;E=0.08;R=83.10;rawP=1.46e-12;adjP=2.99e-12 |
| GnRH signaling pathway | 3845 4893 3265 5894 4313 1956 | C=101;O=6;E=0.04;R=141.76;rawP=2.79e-12;adjP=5.46e-12 |
| Natural killer cell mediated cytotoxicity | 3845 4893 673 369 3265 5894 | C=137;O=6;E=0.06;R=104.51;rawP=1.79e-11;adjP=3.36e-11 |
| B cell receptor signaling pathway | 3845 4893 207 3265 5894 | C=75;O=5;E=0.03;R=159.08;rawP=1.23e-10;adjP=2.20e-10 |
| Chemokine signaling pathway | 3845 4893 673 207 3265 5894 | C=190;O=6;E=0.08;R=75.36;rawP=1.30e-10;adjP=2.20e-10 |
| VEGF signaling pathway | 3845 4893 207 3265 5894 | C=76;O=5;E=0.03;R=156.99;rawP=1.32e-10;adjP=2.20e-10 |
| Fc epsilon RI signaling pathway | 3845 4893 207 3265 5894 | C=79;O=5;E=0.03;R=151.03;rawP=1.61e-10;adjP=2.59e-10 |
| Progesterone-mediated oocyte maturation | 3845 673 369 207 5894 | C=86;O=5;E=0.04;R=138.74;rawP=2.48e-10;adjP=3.85e-10 |
| T cell receptor signaling pathway | 3845 4893 207 3265 5894 | C=108;O=5;E=0.05;R=110.48;rawP=7.91e-10;adjP=1.19e-09 |
| Melanogenesis | 3845 4893 3265 5894 | C=102;O=4;E=0.04;R=93.58;rawP=9.12e-08;adjP=1.32e-07 |
| Cell cycle | 7040 1032 1029 7157 | C=128;O=4;E=0.05;R=74.57;rawP=2.27e-07;adjP=3.19e-07 |
| Tight junction | 3845 4893 207 3265 | C=134;O=4;E=0.06;R=71.23;rawP=2.73e-07;adjP=3.72e-07 |
| Vascular smooth muscle contraction | 673 369 5894 | C=115;O=3;E=0.05;R=62.25;rawP=1.50e-05;adjP=1.99e-05 |
| Axon guidance | 3845 4893 3265 | C=129;O=3;E=0.05;R=55.49;rawP=2.11e-05;adjP=2.71e-05 |
| Dorso-ventral axis formation | 3845 1956 | C=25;O=2;E=0.01;R=190.90;rawP=4.96e-05;adjP=6.20e-05 |
| Endocytosis | 3265 1956 1950 | C=187;O=3;E=0.08;R=38.28;rawP=6.37e-05;adjP=7.75e-05 |
| mTOR signaling pathway | 673 207 | C=52;O=2;E=0.02;R=91.78;rawP=0.0002;adjP=0.0002 |
| Cytokine-cytokine receptor interaction | 7040 1956 1950 | C=267;O=3;E=0.11;R=26.81;rawP=0.0002;adjP=0.0002 |
| p53 signaling pathway | 1029 7157 | C=69;O=2;E=0.03;R=69.17;rawP=0.0004;adjP=0.0005 |
| Apoptosis | 207 7157 | C=88;O=2;E=0.04;R=54.23;rawP=0.0006;adjP=0.0006 |
| Small cell lung cancer | 207 7157 | C=84;O=2;E=0.04;R=56.82;rawP=0.0006;adjP=0.0006 |
| Toll-like receptor signaling pathway | 6696 207 | C=101;O=2;E=0.04;R=47.25;rawP=0.0008;adjP=0.0008 |
| Fc gamma R-mediated phagocytosis | 207 5894 | C=97;O=2;E=0.04;R=49.20;rawP=0.0008;adjP=0.0008 |
| Leukocyte transendothelial migration | 4318 4313 | C=118;O=2;E=0.05;R=40.45;rawP=0.0011;adjP=0.0011 |
